# iTRAQ-Based Comparative Proteomic Analysis of Seedling Leaves of Two Upland Cotton Genotypes Differing in Salt Tolerance

**DOI:** 10.3389/fpls.2017.02113

**Published:** 2017-12-13

**Authors:** Wenfang Gong, Feifei Xu, Junling Sun, Zhen Peng, Shoupu He, Zhaoe Pan, Xiongming Du

**Affiliations:** Institute of Cotton Research, Chinese Academy of Agricultural Sciences, Anyang, China

**Keywords:** cotton, short-term salt stress, salt tolerance, proteome, iTRAQ

## Abstract

Cotton yields are greatly reduced under high salinity stress conditions, although cotton is considered a moderately salt-tolerant crop. Understanding at the molecular level how cotton responds to salt stress will help in developing salt tolerant varieties. Here, we combined physiological analysis with isobaric tags for relative and absolute quantitation (iTRAQ)-based proteomics of seedling leaves of 2 genotypes differing in salinity tolerance to 200 mM (18.3 dS/m) NaCl stress. Salt stress produced significant stress symptoms in the sensitive genotype Nan Dan Ba Di Da Hua (N), including lower relative water and chlorophyll contents and higher relative electrolyte leakage and Na^+^/K^+^ ratio in leaf samples, compared with those in the tolerant genotype Earlistaple 7 (Z). A total of 58 differentially abundant salt-responsive proteins were identified. Asp-Glu-Ala-Asp (DEAD)-box ATP-dependent RNA helicase 3 and protochlorophyllide reductase were markedly suppressed after salt treatment, whereas the phosphate-related differentially abundant proteins (DAPs) phosphoethanolamine N-methyltransferase 1 and 14-3-3-like protein E were induced, and all these proteins may play significant roles in salt stress. Twenty-nine salt-responsive proteins were also genotype specific, and 62.1 and 27.6% of these were related to chloroplast and defense responses, respectively. Based on the *Arabidopsis thaliana* protein interaction database, orthologs of 25 proteins showed interactions in *Arabidopsis*, and among these, a calmodulin protein was predicted to have 212 functional partners. In addition, the Golgi apparatus and calcium may be important for salt secretion in cotton. Through integrative proteome and transcriptome analysis, 16 DAPs were matched to differentially expressed genes and verified using qRT-PCR. On the basis of these findings, we proposed that some proteins related to chloroplast, ATP, ribosomal, and phosphate metabolism as well as to the Golgi apparatus and calcium may play key roles in the short-term salt stress response of cotton seedling leaves.

## Introduction

Salt stress is a major abiotic threat to plants that reduces crop yield (Munns and Tester, 2008). Plants have developed complex adaptive mechanisms in response to salt damage, including adjustment in photosynthesis, synthesis of compatible solutes (glycine betaine, sucrose, and proline), detoxification of reactive oxygen species, and selective ion uptake or exclusion (Tang et al., 2015).

Accumulation of NaCl in cells leads to ionic stresses; excessive amount of NaCl in the environment leads to a competition between Na^+^ and K^+^ transport into the cells, further leading to potassium deficiency (Ghars et al., 2008). K^+^ is important in key metabolic processes in the cytoplasm, such as enzymatic reactions, protein synthesis, and ribosome functions, whereas excessive Na^+^ is toxic to these processes (Shabala and Cuin, 2007). The mechanisms that halophytes use to prevent excessive accumulation of Na^+^ in the cytoplasm include extrusion and/or intracellular compartmentalization of Na^+^ (Peng et al., 2016). To exclude Na^+^ or enable better K^+^ retention in the cytosol, the plasma membrane H^+^-ATPase activity is rapidly upregulated upon salinity treatment in *Atriplex lentiformis* and *Chenopodium quinoa* (Bose et al., 2014). Many genes associated with the extrusion and/or intracellular compartmentalization of Na^+^, such as *Arabidopsis thaliana Na*^+^*/H*^+^ *antiporter 1*(*AtNHX1*), *Sorghum bicolor Na*^+^/*H*^+^ *antiporter-like protein* (*SbNHXLP*), *A. thaliana salt overly sensitive 1*(*AtSOS1*), *A. thaliana salt overly sensitive 2*(*AtSOS2*), *Arabidopsis vacuolar pyrophosphatase 1* (*AVP1*), and *Osmotin like protein* (*OLP*), have been identified (Guo et al., 2012; Kumar et al., 2015, 2016; Peng et al., 2016; Kumari et al., 2017). *SbNHXLP*, a new member of the *Na*^+^*/H*^+^ *antiporter* (*NHX*) gene family, is not only involved in excluding Na^+^ from the cytoplasm but also in acquiring K^+^ and maintaining a high K^+^/Na^+^ ratio in transgenic tomato. Overexpression of *SbNHXLP* confers salt tolerance in tomato by maintaining ion homeostasis (Kumari et al., 2017). It is worth mentioning that Osmotin like protein belonging to the pathogenesis-related (PR)-5 family also plays an important role in salt stress tolerance by sequestering Na^+^ ions and compartmentalizing them into vacuoles and intercellular spaces (Kumar et al., 2015, 2016).

Photosynthesis, among the primary processes affected by salinity, is extremely sensitive to salt stress (Munns et al., 2006). Salinity has various effects on basic processes of photosynthesis, such as photosynthetic pigment synthesis, electron transport reactions, photophosphorylation, and CO_2_ fixation (Silveira and Carvalho, 2016). Several proteomics reports have shown that upon exposure to salt stress, light reaction-related proteins (Oxygen-evolving enhancer protein 1, Oxygen-evolving enhancer protein 2, and chloroplast manganese stabilizing protein) present significant differences in abundance (El Rabey et al., 2016; Silveira and Carvalho, 2016). Calvin cycle-related proteins, such as Ribulose bisphosphate carboxylase/oxygenase (RuBisCO) activase and phosphoglycerate kinase, were observed in abundance (Wang et al., 2015). The abundance of cytochrome b6f complex, which transfers electrons from Photosystem II (PSII) to Photosystem I (PSI), is also affected by salt stress (Zhang et al., 2012); however, the detection of modulation of proteins related to PSI is less frequent than that of those related to PSII among plant responses to salt stress due to proteomics technique limitations (Silveira and Carvalho, 2016). Different species exhibit different relative limitations to photosynthesis under stress. In *Thellungiella* (a stress-tolerant plant), photosynthesis genes constitute the largest functional group of downregulated transcripts (15%) (Wong et al., 2006), whereas in rice, alterations in photosynthesis-related genes are mostly associated with stress recovery (Zhou et al., 2007).

High-throughput sequencing is a powerful tool for identifying salt tolerance-related genes, studying complex networks of physiological and developmental gene interactions, and further elucidation of salt tolerance mechanisms. Many high-throughput transcriptome data sets have identified many transcriptional changes to date (Diray-Arce et al., 2015). Due to post-translational modifications, mRNA levels cannot be generally correlated with protein levels (Maier et al., 2009). Therefore, the use of proteome analyses, which can identify proteins involved in the salt-stress response, has become an important strategy. More than 2,171 salt-responsive proteins have been identified in many tissues from 34 plant species (Zhang et al., 2012). Widely expanded proteomics approaches have been integrated with bioinformatics advances, generating favorable outcomes for the elucidation of complex mechanisms involved in salt stress effects.

Cotton (*Gossypium hirsutum* L.), an important cash crop that produces both fiber and oil, is considered to be a moderately salt-tolerant crop (Ashraf, 2002). According to Basal et al. (2006), when exposed to 150 and 200 mM NaCl, 3 of 10 genotypes may provide additional insights and parental material for breeding salt-resistant cottons. Du et al. (2012) found that 7 cotton varieties can tolerate up to 300 mM NaCl. However, with the reduction in field areas, the competition between food crops and cotton has become prominent and cotton is increasingly planted in saline soils. Therefore, it is of great importance to identify salt tolerance-related genes and proteins and to elucidate salt tolerance mechanisms in cotton. A cotton dehydration responsive element (DRE)-binding transcription factor called *G. hirsutum dehydration responsive element binding* (*GhDREB*) gene exhibits tolerance to high salt stress in transgenic wheat, as reported in a study conducted by Gao et al. (2009). Six genes from the NAM-ATAF1/2-CUC (NAC) gene family (*GhNAC1–GhNAC6*) have also been isolated from cotton, and they were differentially regulated under conditions involving drought, high salt, and other abiotic stresses (Meng et al., 2009). Many high-throughput transcriptomic studies on salt stress have identified salt tolerance-associated candidate genes, such as *WRKYY transcription factors* (Proteins in this family contain the conserved WRKYGQK amino acid sequence at the N-terminus)*, ethylene responsive element binding factors* (*ERFs*), and *jasmonate-ZIM-domain protein* (*JAZ*) gene (Yao et al., 2011; Zhang et al., 2011, 2013). A comparative proteome analysis of cotton fibers identified 132 drought-responsive proteins that were differentially accumulated (Zheng et al., 2014). Other proteomic analyses of fiber development and evolution in cotton have also been reported (Du et al., 2013; Hu et al., 2013). However, proteomic studies on cotton leaves under salt stress are very limited, and studies on different cotton genotypes with different degrees of salt tolerance are even rarer. Cui et al. (2012) identified 4 differentially abundant proteins (DAPs) in the leaves of one salt-tolerant cotton variety that was subjected to 0.4% NaCl for 24 h. In addition, long-term treatments tend to be used for protein profiling in response to salt stress; thus, little information is available about the initial phase of salt stress. Plants adapted to long-term salt stress reveal different physiological responses triggered by osmotic stress as well as ion toxicity and a marked change in the protein pattern; thus, a change in the protein pattern indicated more general stress mitigation rather than a specific salt-dependent reaction. In addition, some proteins such as coupling factor 1 epsilon (CF1e) of maize only showed a rather transient response in the initial phase of salt stress (Zörb et al., 2009). Therefore, to enrich and optimize the salt-responsive proteome, it is important to study the proteome dynamics of salt-tolerant and -sensitive cotton genotypes, particularly under short-term treatment.

**Figure 1:**
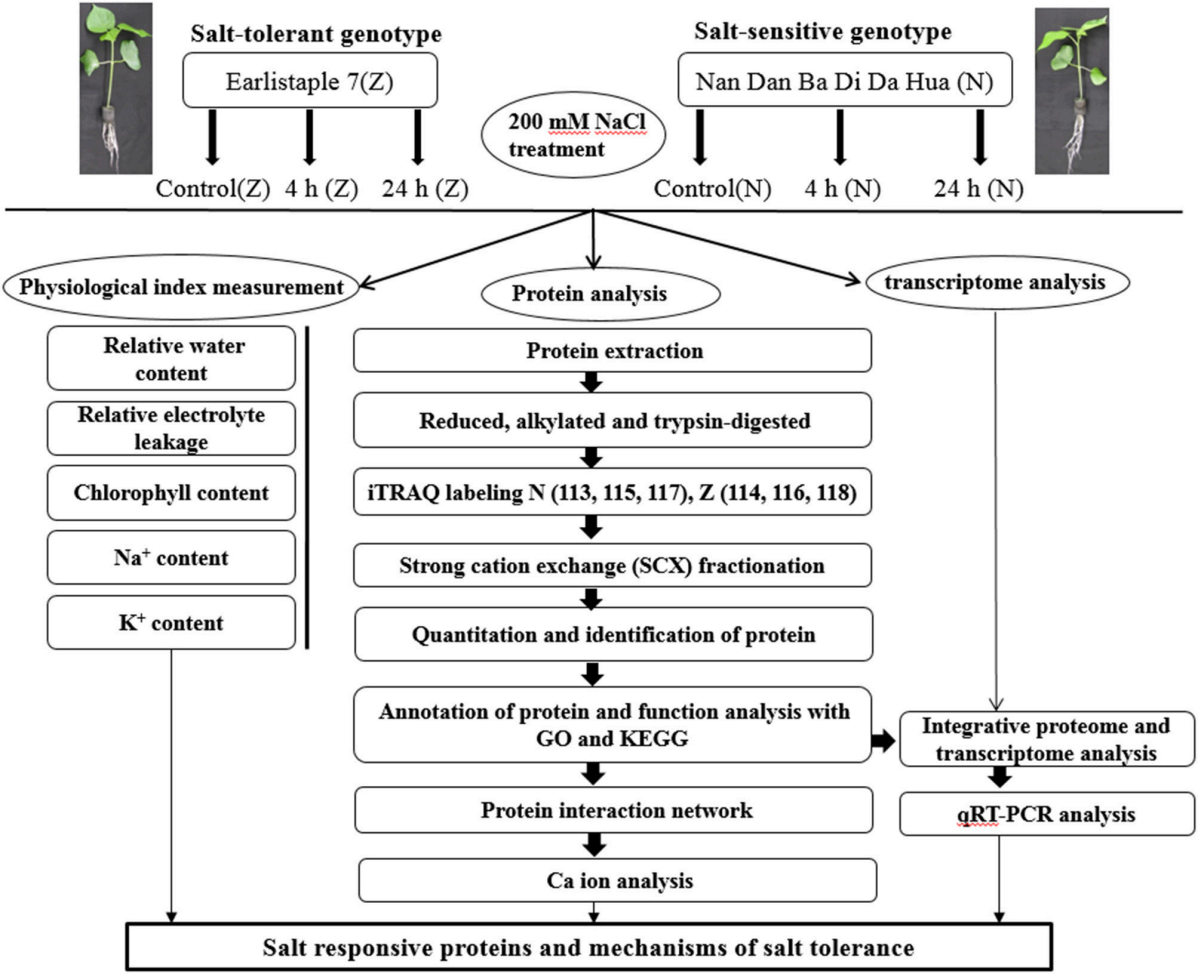
Schematic representation of the experimental design. The main objective of this study was to identify salt responsive differentially abundant proteins to develop a better understanding of the mechanisms of salt stress tolerance in cotton. Seedlings at the 3-leaf stage were treated with 200 mM (18.3 dS/m) NaCl for 4 h, and 24 h. The plants without salt stress were considered as control. Then the leaves of treated seedlings were collected and prepared for physiological studies and proteome analysis. All the sample collections and preparations were repeated in triplicate, and each independent biological experiment has 3 technological replicates. Physiological tests were conducted just after leaf collection using fresh cotton samples. The transcriptome analysis was performed by Peng et al. (2014). For proteome analysis, the protein extracts were digested using trypsin, and peptides were labeled with isobaric tags for relative and absolute quantitation (iTRAQ) reagents. The numbers 113, 114, 115, 116, 117, and 118 were different iTRAQ labels. Labeled peptides were fractionated by strong cation exchange chromatography and analyzed using LC-MS/MS. Data analysis was performed with Proteome Discover 1.3 and Mascot software 2.3.02. *Arabidopsis thaliana* protein interactome database (AtPID) served the purpose in finding orthologous proteins of interactors found in *Arabidopsis*. As depicted in our findings, we can propose that some proteins related to chloroplast, ATP, ribosomal, and phosphate metabolism as well as to the Golgi apparatus and calcium might denote important mechanisms for the short-term salt stress response of cotton seedling leaves.

Two upland cotton varieties, the salt-sensitive genotype Nan Dan Ba Di Da Hua (N) and the salt-tolerant genotype Earlistaple 7 (Z), were identified after growth under salt stress (Du et al., 2012). In the present study, we aimed to identify DAPs associated with salt tolerance and to highlight the potential response mechanism of salt stress in cotton seedling leaves. The isobaric tags for relative and absolute quantitation (iTRAQ)-based proteomic analyses of 2 genotypes (N and Z) showing different tolerances to salt stress were performed. In addition, the physiological changes induced by salt stress were observed for each genotype. Moreover, we compared the changes at the proteomic and transcriptional levels under the same salt stress conditions. The workflow was set out in Figure 1. The result is expected to provide important insights into physiological and molecular mechanisms associated with salt tolerance in upland cotton seedling leaves.

## Materials and methods

### Sample collection and preparation

The salt-tolerant upland cotton genotype Z and the salt-sensitive genotype N were used in this experiment. N was introduced from Guangxi province, China, before 1978, whereas Z was selected from Earlistaple 808 introduced from Pee Dee Experiment station, Florence, America in 1982. Both of these were kept in the National Mid-term gene bank for cotton in China (Du et al., 2012). The seeds of both genotypes were sterilized with 70% (v/v) ethanol, 0.1% (w/w) mercury chloride, and 0.2% (w/w) thiram. They were then washed 4 times with sterilized water, submerged in water for 12 h at room temperature, and then sown in vermiculite for germination. After 4 days, the seedlings were transferred to hydroponic containers containing modified Hoagland solution for germination. The seedlings were then grown in a growth chamber with a 14-h light/10-h dark cycle, 28°C/22°C day/night temperature, 450 μmol/m^2^/s light intensity, and 60–80% relative humidity. Seedlings at the 3-leaf stage were treated with 200 mM (18.3 dS/m) NaCl for 4 and 24 h. For Ca^2+^ content measurement, seedlings were exposed to 200 mM NaCl for 4, 8, 24, 48, and 72 h. The plants without salt stress were considered as control. The 3 leaves of treated seedlings were all collected, mixed uniformly, and prepared for physiological studies and proteome analysis. Physiological tests were conducted just after leaf collection using fresh cotton samples; the other samples were placed in liquid nitrogen and stored at −80°C. All sample collection and preparation procedures were performed in triplicate, and each independent biological experiment has 3 technological replicates.

### Measurement of the relative water content (RWC), relative electrolyte leakage (REL), chlorophyll content, Na^+^ content, K^+^ content and Ca^2+^ content

RWC and REL were tested and calculated as previously described (Cao et al., 2007; Katam et al., 2016). For RWC, leaf fresh weight (FW) was measured immediately after sample collection, and the sample was then left to saturate in water for 8 h at 4°C before measuring the turgid weight (TW). The samples were oven dried at 80°C for 24 h and weighed (DW). RWC was calculated as follows: RWC = (FW – DW)/ (TW – DW).

For REL, 0.5 g of fresh leaves were cut into discs 0.8 cm in diameter, placed in 40 mL of ddH_2_O, and incubated at room temperature for 4 h. Then, electrical conductivity of the solution (C1) was measured using a conductivity meter (EM38, ICT international, Armidale, NSW, Australia). The solution was boiled for 10 min and cooled to room temperature, and electrical conductivity (C2) was again measured. REL was calculated as REL = C1/C2.

For chlorophyll content, 0.2 g of fresh leaves was incubated in 20 mL of 80% acetone in the dark at 4°C overnight. After centrifugation at 5,000 × g and 4°C for 5 min, absorbance of the supernatant was measured at 663 and 645 nm using a spectrophotometer (DU 800, Beckman, Coulter, Inc., 250 S. Kraemer Boulevard Brea, CA 92821, USA). Chlorophyll content was calculated as total chlorophyll content = 2.03 D645 + 0.804 D663.

To measure Na^+^, K^+^, and Ca^2+^ content, fresh tissues were washed with distilled water immediately after collection, dried at 60°C for 72 h, and ground into a fine powder using a mortar and pestle. Approximately 200–500 mg of powder from each genotype was added to 12 mL of 65% HNO_3_ and 2 mL of 30% H_2_O_2_ and incubated at 80°C for 1 h. Na^+^, K^+^ and Ca^2+^ concentrations of the leaves were determined by inductively coupled plasma-optical emission spectrometry (Optima 2100 DV; Perkin-Elmer, Inc., Massachusetts, USA) according to the manufacturer's instructions.

### Protein extraction, digestion, and iTRAQ labeling

Leaves were ground into a powder under liquid nitrogen and suspended in extraction buffer (v/v = 1:3) containing precooled acetone (−20°C), 10% (w/v) Trichloroacetic acid (TCA), and 0.07% (v/v) 2-mercaptoethanol. Initially, the proteins were precipitated for 2 h at −20°C after vortexing and then collected by centrifugation at 14,000 × g for 60 min. After careful removal of the supernatant, protein pellets were washed twice with cold acetone containing 0.07% 2- mercaptoethanol, dried using a vacuum freeze dryer for 2 h, and immediately extracted using protein extraction buffer consisting of 7 M urea, 2 M thiourea, 40 mM Dithiothreitol (DTT), 4% 3-[(3-Cholamidopropyl) dimethylammonio]-1-propanesulfonate (CHAPS), 1% protease inhibitor cocktail, and 2% Pharmalyte 3-10 for 2 h at 4°C under continuous shaking. In addition, proteins were centrifuged at 100,000 × g for 60 min at 4°C. Finally, the supernatant was collected and protein concentration was determined using a 2-D Quant Kit (General Electric Company, USA). Sodium dodecyl sulfate polyacrylamide gel electrophoresis (SDS-PAGE) (12% gels) was used to verify the protein quality and concentration. A total of 30 μL of the protein sample was added to the gel. Three biological replicates were used (Supplementary Figure S1). For each protein sample, 100 μg of proteins were reduced, alkylated, trypsin digested, and labeled using the iTRAQ Reagent 4-plex Kit according to the manufacturer's instructions (Applied Biosystems, Foster City, CA). N samples were labeled with the iTRAQ tags 113, 115, and 117, whereas Z samples were labeled with the iTRAQ tags 114, 116, and 118. Three independent biological experiments with 3 technological replicates were performed.

### Strong cation exchange (SCX) fractionation and liquid chromatography coupled with tandem mass spectrometry (LC-MS/MS)

SCX and LC-MS/MS were performed as previously described with minor modifications (Qin et al., 2013). The SCX protocol was modified by changing the elution buffer: buffer A (25 mM NaH_2_PO_4_ in 25% acetonitrile (ACN), pH 2.7) and buffer B (25 mM NaH_2_PO_4_ and 1 M KCl in 25% ACN, pH 2.7). The following gradient was also modified: buffer A for 10 min, 5–35% buffer B for 11 min, and 35–80% buffer B for 1 min. The system was then maintained in 80% buffer B for 3 min before equilibrating with buffer A for 10 min. LC-MS/MS parameters included the following: analytical separation solvents comprising acetonitrile/formic acid (A: 2/0.1%; B: 98/0.1%); 2.25 μg of each sample was loaded at 15 μL/min for 4 min and then the 44 min gradient was run at 400 nL/min starting from 2 to 35% B, followed by the 2 min linear gradient to 80% B and 4 min maintenance at 80% B, and subsequent 1 min return to 2% B. With a TripleTOF 5600 System (AB SCIEX, Concord, ON), data were acquired in 250 ms, and as many as 30 product ion scans were collected if a threshold of 120 counts per second was exceeded, with a 2+ to 5+ charge state and a 18-s dynamic exclusion setting.

### Database search and quantification

Raw mass data were processed with Proteome Discover 1.3 (Thermo Fisher Scientific) and searched with Mascot software 2.3.02 (Matrix Science, London, U.K.) against the database downloaded from ftp://ftp.ncbi.nlm.nih.gov/genomes/genbank/plant/Gossypium_raimondii/latest_assembly_versions/. The search parameters were set as follows: trypsin was specified as the digestion enzyme, carbamidomethylation of cysteine was set as a fixed modification, oxidation of methionine was set as a variable modification, peptide tolerance was set to 10 ppm, and MS/MS tolerance was set to 0.05 Da. Proteins with at least 2 unique peptides and a threshold *p*-value (with 95% confidence) of < 0.05 were qualified for further quantitative data analysis. For protein abundance ratios, fold-changes of ≥1.2 (or ≤0.83) and *p*-values of < 0.05 were taken as thresholds to identify significant changes.

### Gene ontology (GO), pathway enrichment, and cluster analysis

Functional annotation of the proteins was conducted with the Blast2GO (http://www.geneontology.org) program against the non-redundant protein database (NR; National Center for Biotechnology Information). The GO enrichment terms of DAPs shortlisted with above criteria were identified by the hypergeometric test (*P* < 0.05). Pathway enrichment analysis of significant proteins was performed using the Kyoto Encyclopedia of Genes and Genomes (KEGG) database (http://www.genome.jp/kegg/). The pathway of significant enrichment compared with differential proteins tested with *P* < 0.05 was used as a threshold to select significant KEGG pathways.

### Measurement of enzyme activity

The sucrose synthase assay was performed at 290 nm using sucrose synthetase assay kit acquired from Nanjing Jiancheng Bioengineering Research Institute (Nanjing, China) according to the manufacturer's instructions.

For estimating the activity of glutathione *S*-transferase, 0.5 g seedlings leaves were homogenized in 1 mL of ice cold extraction buffer and determined by measuring the formation of the conjugate reaction product at 340 nm using 1-chloro-2,4-dinitrobenzene and glutathione as substrates according to the method of Venisse et al. (2001).

The fructose 1,6 bisphosphate aldolase activity was determined using fructose 1,6 bisphosphate aldolase assay kit (Suzhou Comin Biotechnology Co. Ltd., Suzhou, China) by observing the change in absorbance at 340 nm for 5 min according to the manufacturer's instructions.

### Protein–protein interaction analysis

According to Katam et al. (2010, 2016), *A. thaliana* protein interaction database (AtPID) has been used to study the protein–protein interaction in cotton leaves. Protein orthologs were identified in *Arabidopsis* using UniProt and The Arabidopsis Information Resource.

### Real-time RT-PCR

Total RNA was isolated from cotton leaf samples treated with either 200 mM NaCl or water (as a control) for 4 or 24 h. For cDNA synthesis, 500 ng of total RNA for each sample was used for reverse transcription using the PrimeScript™ RT Reagent Kit with gDNA Eraser (TaKaRa, Japan) according to the manufacturer's instructions. Real-time RT-PCR was performed using the SYBER premix ExTaq Kit (TaKaRa, Japan) and the 7500 Real-Time PCR system (Applied Biosystems). *GhActin* (AY305733) was used as the reference gene. The primers are listed in Supplementary Table S1. The relative expression level of each gene was calculated by the 2^−ΔΔCt^ method (Schmittgen and Livak, 2008). Three independent biological experiments with 3 technological replicates were performed.

### Statistical analysis

All the data were subjected to statistical analysis with SPSS 19.0 software (IBM Corp, Armonk, NY, USA). Physiological data were analyzed using Student's *t*-test and the Tukey method for one-way ANOVA at 95% confidence. Real-time RT-PCR results were evaluated with the Student's *t*-test. The data were expressed as the means ± standard errors. For all the analyses, a *P*-value of < 0.05 indicated statistical significance.

## Results

### Physiological changes under salt stress

**Figure 2:**
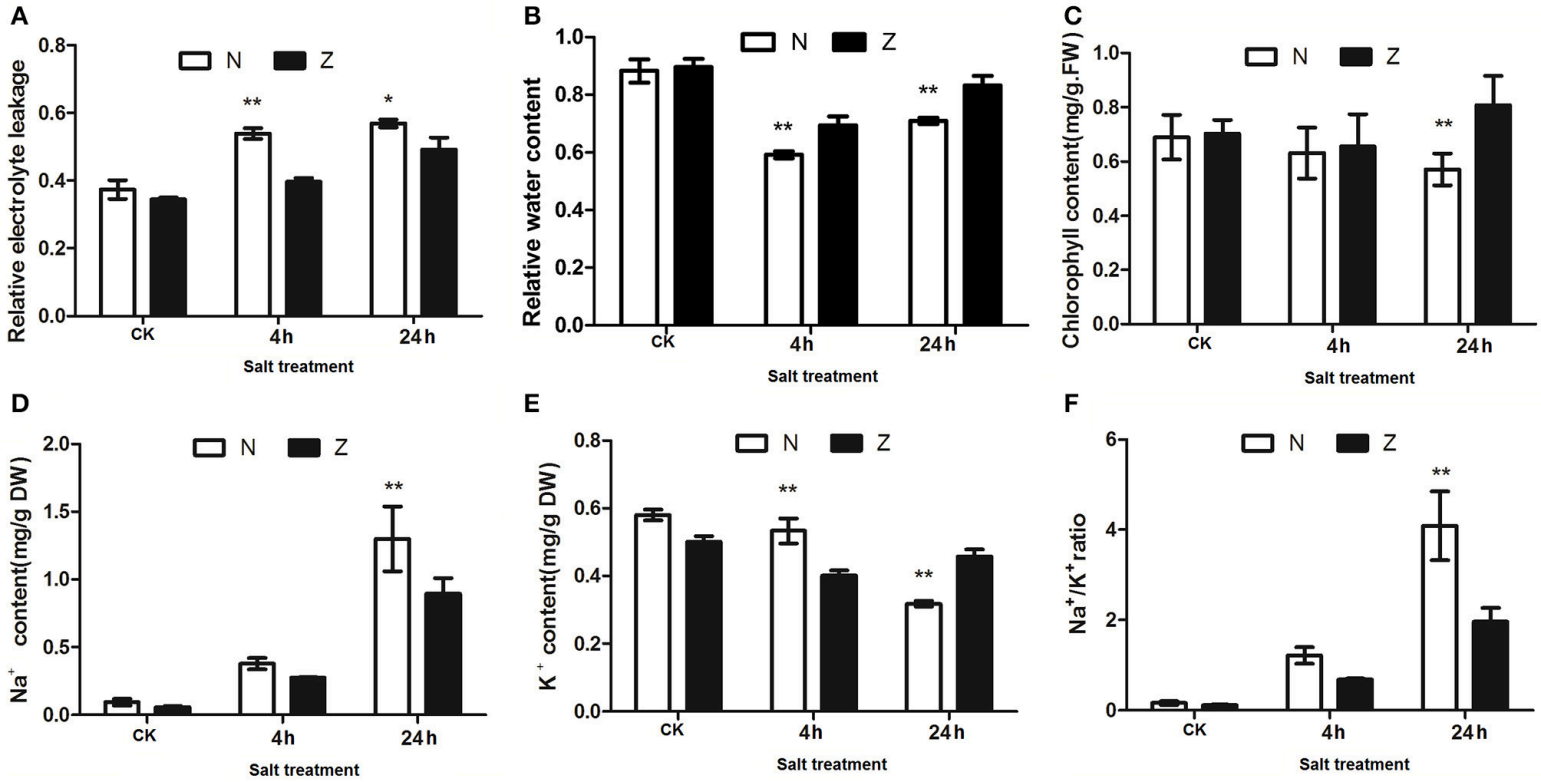
Physiological changes in the salt treatment of seedlings from 2 cotton varieties, N and Z. Effects on **(A)** relative electrolyte leakage, **(B)** relative water content, **(C)** chlorophyll content, **(D)** Na^+^ content, **(E)** K^+^ content, and **(F)** Na^+^/K^+^ ratio of the third true leaves in N and Z subjected to 200 mM NaCl. The results were the means ± standard errors of 3 biological replicates (each of which has 3 technical replicate treatments, ^*^P < 0.01, ^**^P < 0.001). The white and black columns represented the N and Z values, respectively.

Four physiological indices were employed to detect the impact of salinity stress on cotton physiology. Under stress, N displayed higher REL compared with Z after 4 and 24 h. (Figure 2A). The NaCl treatment resulted in an initial decrease and then an increase in RWC in both the N and Z genotypes; however, the water content was observed to be comparatively higher in Z (Figure 2B). Chlorophyll concentration was significantly higher in Z than that in N after 24 h of salt stress (Figure 2C). The Na^+^ content in N showed a similar trend as that in Z, and in both of these genotypes, it continuously increased after 4 and 24 h of salt stress. However, the concentration was significantly higher in N than that in Z at 24 h (Figure 2D). The K^+^ content in Z decreased at first and then increased, and it consistently decreased, particularly at 24 h, in N. Compared with N, the K^+^ content in Z was lower at 4 h but higher at 24 h (Figure 2E). The Na^+^/K^+^ ratio increased in both genotypes compared to that in the non-salt stress controls, with the ratio being much higher in N than that in Z, particularly at 24 h (Figure 2F).

### Identification of DAPs using iTRAQ LC-MS/MS

A total of 6,338, 6,347, and 6,376 peptides and 5,472, 5,452, and 5,451 unique peptides with 2,356, 2,334, and 2,325 non-redundant protein groups in 3 biological replicates were identified in cotton seedlings treated with 200 mM NaCl. They were identified at a 95% confidence level and a 0.68% false discovery rate. GO analysis of the identified proteins showed that most proteins were metabolic processes and binding related (Supplementary Figure S2). To obtain differentially abundant proteins (DAPs), 7 comparison schemes N4/Nck, N24/Nck, Z4/Zck, Z24/Zck, Zck/Nck, Z4/N4, and Z24/N24 were set (N4 and N24 represent N seedlings subjected to 200 mM NaCl for 4 and 24 h, respectively; Z4 and Z24 represent Z seedlings subjected to 200 mM NaCl for 4 and 24 h, respectively; and Nck and Zck were the control plants without salt treatment). For greater confidence, only proteins with 2 or more peptide matches were screened for subsequent analysis. The proteins with ratio values of more than 1.2 or less than 0.83 (*P* < 0.05) were defined as DAPs. A total of 115 DAPs in the above 7 comparison schemes were identified in all 3 replicates (Supplementary Table S2).

GO and KEGG analyses for the DAPs were performed to explore the possible roles of these proteins in salt stress. For biological processes, the most common terms were response to stimulus and single-organism metabolic process, and photosynthesis was the most prominently enriched GO term (Supplementary Figure S3A). For the cellular component category, cytoplasmic part and intracellular organelle part were the most frequent categories; chloroplast part and plastid part were the most significant terms (Supplementary Figure S3B). The top 20 terms, including bind, ion binding, cation binding, oxidoreductase activity, structural molecular activity and structural constituent of ribosome, were significantly enriched in the molecular function class (Supplementary Figure S3C). A KEGG pathway analysis showed that these DAPs were primarily participated in porphyrin and chlorophyll metabolism, glyoxylate and dicarboxylate metabolism, photosynthesis and ribosome (Supplementary Figure S3D).

A total of 58 salt-responsive DAPs (Supplementary Table S3) were present in the 4 comparisons (N4/Nck, N24/Nck, Z4/Zck, and Z24/Zck) in both N and Z under the 200 mMNaCl treatment. Of these DAPs, 65.5, 50.0, 25.9, 24.1, 17.2, 15.5, 13.8, 3.4, and 1.7% were related to chloroplast, membrane, ion, phosphate, ATP, cadmium, Golgi, calcium, and endoplasmic reticulum, respectively (Supplementary Figure S4A). To determine the differential abundance pattern of salt-responsive DAPs, 6 clusters were generated. Group 1 contained 1 protein that decreased in both genotypes after 4 and 24 h of salt treatment. Group 2 included 3 proteins, which increased in both genotypes after 4 and 24 h of salt treatment. Four proteins were classified as Group 3, which were abundant at 4 h, and 3 of these were ribosomal proteins. Group 4, which included 7 proteins, showed a marked downward trend at 24 h. Group 5 included 13 proteins with an upward trend at 24 h. The other proteins, which have differential response between Z and N, were classified into Group 6 (Supplementary Figure S5, Supplementary Table S3).

**Figure 3:**
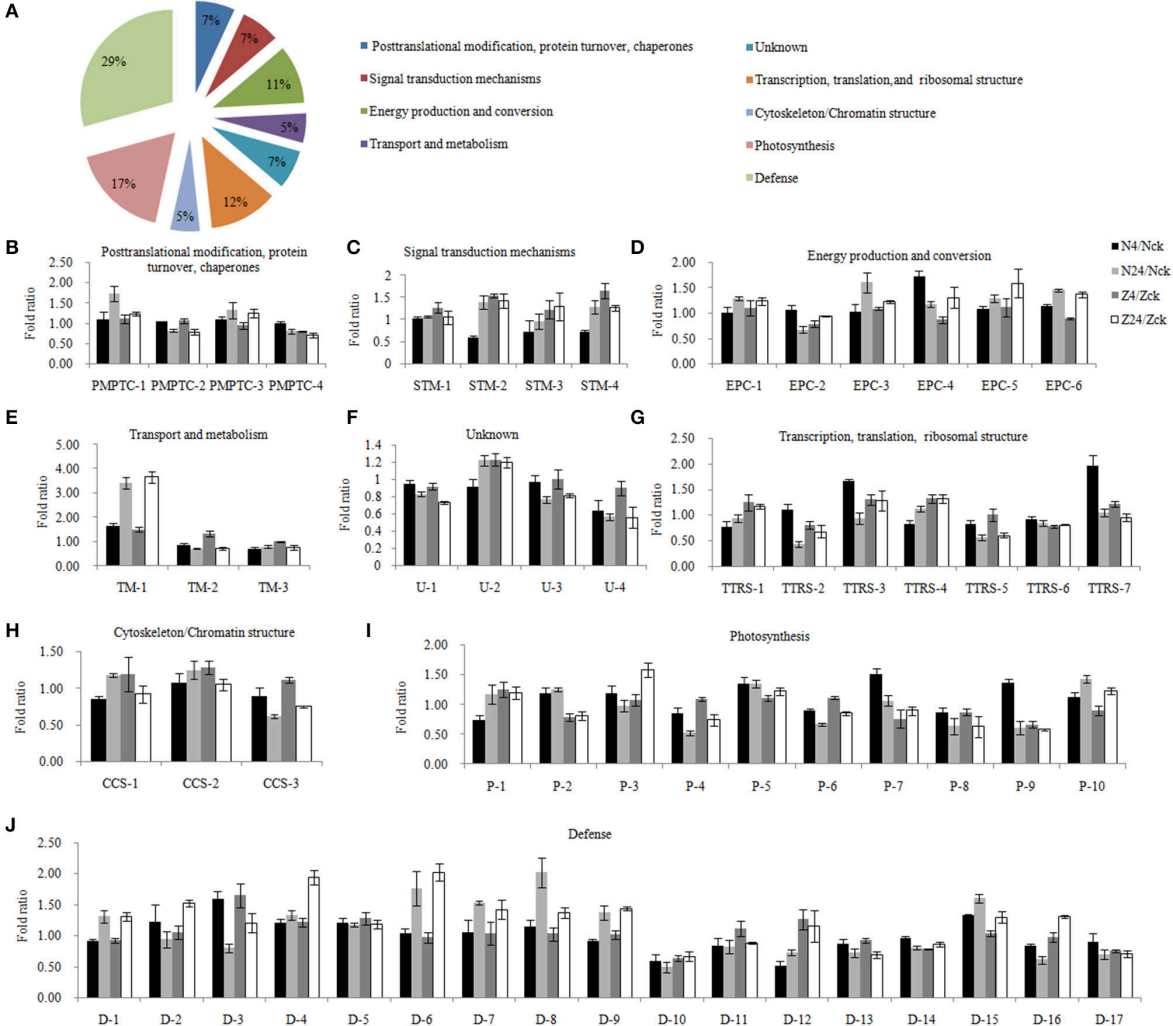
Functional classification and the patterns of dynamic response of 58 proteins to different NaCl concentrations. **(A)** Functional cataloging of salt stress responsive differentially abundant proteins. The abundance fold ratio of 58 proteins involved in post-translational modification, protein turnover, chaperones category **(B)**, signal transduction mechanisms **(C)**, energy production and conversion **(D)**, transport and metabolism **(E)**, unknown **(F)**, transcription, translation, and ribosomal structure **(G)**, cytoskeleton/chromatin structure **(H)**, photosynthesis **(I)**, and defense **(J)**. The results were the means ± standard errors of 3 biological replicates (each of which has 3 technical replicates). The details were provided in Supplementary Table S4. N4/Nck, N24/Nck, Z4/Zck and Z24/Zck are 4 comparison sets of 2 genotypes under 200 mM NaCl stress for 4 and 24 h. N, salt-sensitive genotype Nan Dan Ba Di Da Hua; Z, salt-sensitive genotype Earlistaple 7.

To elucidate the regulatory pathways, these DAPs were classified into 9 functional categories (Figure 3A, Supplementary Table S4). Four proteins were grouped into the post-translational modification, protein turnover, chaperones category; all these did not show significant changes in protein abundance after 4 h but showed abundance or low abundance after 24 h of salt stress (Figure 3B). In the signal transduction mechanism category, 3 of 4 proteins were abundantly accumulated (probable ribose-5-phosphate isomerase, 14-3-3-like protein E, and calmodulin-1/11/16) (Figure 3C). The energy production and conversion group included 6 proteins (Figure 3D). Phosphoethanolamine N-methyltransferase 1, which belongs to the transport and metabolism category, showed the highest abundance (Figure 3E). The functional category of 4 proteins was unknown (Figure 3F). Seven proteins were related to transcription, translation and ribosomal structure; only 3 proteins showed low abundance after 24 h, including Asp-Glu-Ala-Asp (DEAD)-box ATP-dependent RNA helicase 3, which showed low protein abundance in both N and Z, and 40S ribosomal protein S10, which showed a marked change in abundance after salt stress (Figure 3G). The cytoskeleton/chromatin structure contained 3 proteins (Figure 3H). Ten proteins were associated with photosynthesis. PSI reaction center subunit III abundance was unaffected after 4 h but highly increased after 24 h; however, oxygen-evolving enhancer protein 3 abundance showed a downward trend after salt stress (Figure 3I) Our data also demonstrated that more than 29.3% (17 proteins) of the salt-responsive proteins were involved in defense responses, such as peroxidase 15, glutathione S-transferase F9, and pathogenesis-related protein STH-2 (Figure 3J).

Furthermore, 63 genotype-specific DAPs (Supplementary Table S5) were present in only N or Z or in both N and Z (Zck/Nck, Z4/N4, and Z24/N24) but with different abundance. Of these DAPs, 65.1, 17.5, 14.3, 12.7, 6.3, and 6.3% were related to the chloroplast, phosphate, ATP, Golgi, calcium, and endoplasmic reticulum, respectively (Supplementary Figure S4B). As shown in Supplementary Figure S6 and Supplementary Table S5, these proteins were clustered in 6 groups. Group 1 included 7 proteins, which showed low abundance in Z compared with N without salt stress but high or low abundance under stress. On the contrary, before salt treatment, 5 proteins in Group 2 showed high abundance in Z compared with N but abundance or low abundance after salt treatment. Group 3 (9 proteins) showed significant differences between the 2 genotypes in controls but was unaffected under salt stress. Groups 4, 5, and 6 included proteins that showed no significant differences between these 2 genotypes without salt stress but changed upon exposure to NaCl stress. After treatment with 200 mM NaCl, 13 proteins in Group 4 showed high or low abundance at 4 and 24 h, whereas 13 proteins in Group 5 showed increased or decreased abundance at 4 h, and 16 proteins in Group 6 showed change in abundance at 24 h. As shown in Supplementary Figure S7 and Supplementary Table S6, these genotype-specific DAPs were classified into the same 9 functional categories, and the majority of proteins were clustered to defense responsive groups.

**Figure 4:**
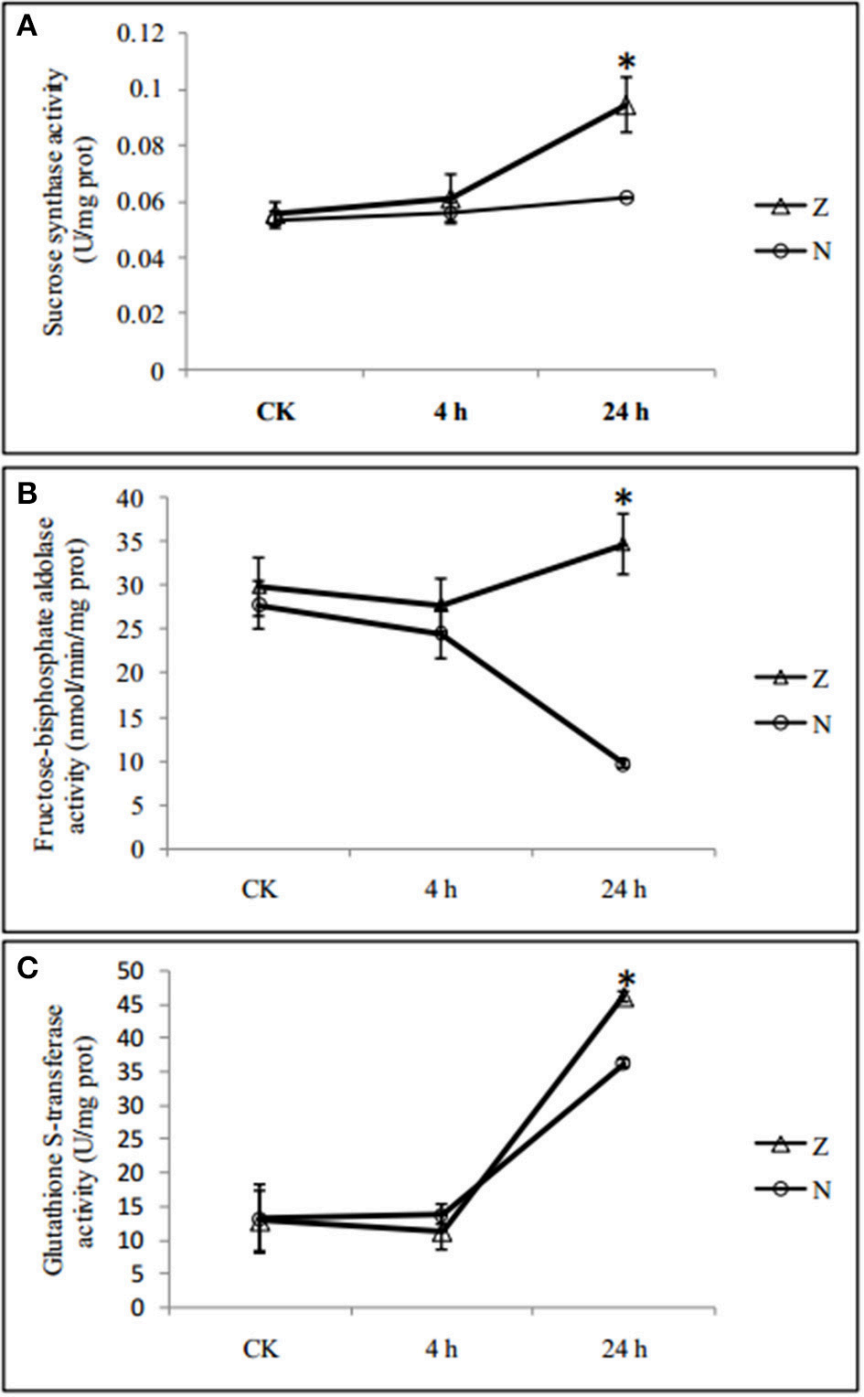
Salinity induced changes in activities of enzymes in seedlings leaves from 2 cotton varieties, N and Z. The activities of **(A)** Sucrose synthase, **(B)** Fructose 1,6 bisphosphate aldolase, and **(C)** Glutathione S-transferase were measured in seedlings leaves from 2 cotton varieties, N (circle) and Z (triangle) treated with 200 mM NaCl for 4 and 24 h. Values were the means ± standard errors of 3 biological replicates (each of which has 3 technical replicate treatments, ^*^P < 0.05).

Among the 58 salt-responsive DAPs, 29 proteins did not show genotype specificity (common salt-responsive DAPs), including highly abundant phosphoethanolamine N-methyltransferase 1 and 14-3-3-like protein and low abundant DEAD-box ATP-dependent RNA helicase 3 and protochlorophyllide reductase, after salt treatment. Another 29 salt-responsive DAPs were also genotype specific (Supplementary Table S7). Eight (27.6%) of these were associated with defense response, 18 (62.1%) proteins were related to chloroplast, and 11 proteins (37.9%) were related to ion binding or hydrogen ion transmembrane transporters, including calcium ion binding related proteins. The changes in protein abundance were further validated by enzyme activity assay. In agreement with the data from the iTRAQ analysis, the activities of sucrose synthase and glutathione s-transferase were significantly increased at 24 h of salt stress treatment in both genotypes, respectively, while the fructose 1,6 bisphosphate aldolase activity was significantly decreased in N but increased in Z at 24 h (Figure 4).

**Figure 5:**
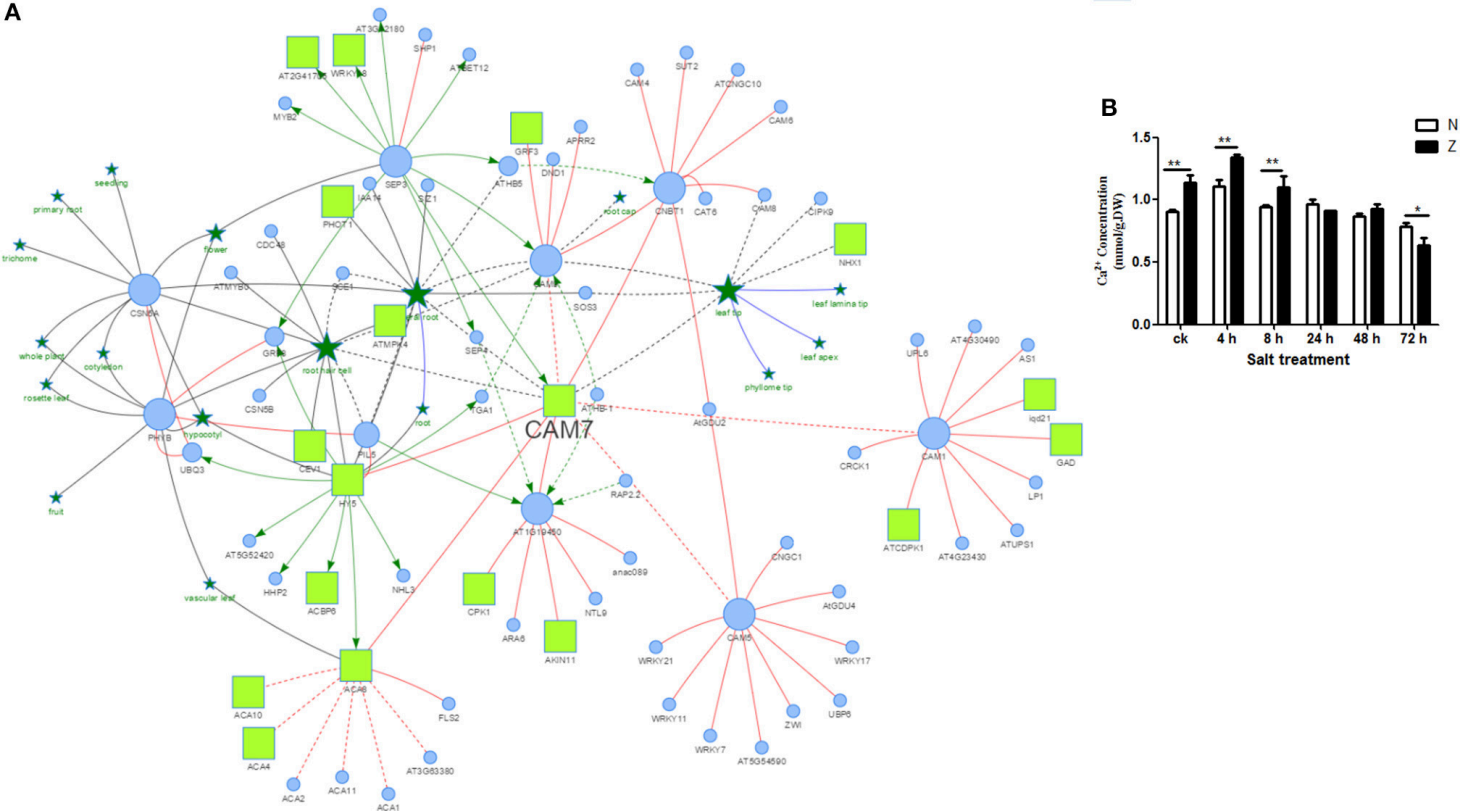
Possible protein interactions of a calmodulin protein CAM7 derived using the *Arabidopsis thaliana* Plant Interaction Database **(A)** and Ca^2+^ content measurement of cotton seedling leaves exposed to 200 mM NaCl **(B)**. 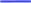: PPI; 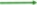: PO-PO association; 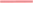: PO-gene association; 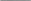: Transcriptional regulation; 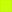: Protein; 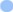: Plant Ontology entry; 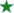: Phosphoprotein; 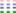: Predicted information; 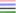: Authentic information with literature evidence; The node size means the degree of the node. The results were the means ± standard errors of 3 biological replicates (each of which has 3 technical replicate treatments, ^*^P < 0.05, ^**^P < 0.01). The white and black columns represented the N and Z values, respectively.

As reported by Katam et al. (2010, 2016), AtPID was used to analyze the interaction of these 29 genotype-specific salt-responsive DAPs. Orthologs of 25 proteins showed interactions in *Arabidopsis*. Among these, AT3G43810.1, a calmodulin protein, had the largest number of functional partners (212 proteins) (Figure 5A, Supplementary Table S8).

Salt stress was found to significantly affect the abundance of calcium related proteins (Supplementary Figure S4, Supplementary Tables S4, S6, S8). To further analyze the effect of salt stress on calcium, the Ca^2+^ concentration was measured (Figure 5B) in the seedling leaves. After exposure to the salt stress, the Ca^2+^ concentration in Z showed a similar trend as that in N; both of them increased at first (4 h) and then decreased continuously. The Ca^2+^ content was significantly higher in Z than that in N before 8 h; however, it was not significantly different at 24 h and 48 h. At 72 h, it was even higher in N than that in Z. Taken together; we speculated that calcium might play an important role in the salt tolerance of cotton.

### Integrative proteome and transcriptome analysis during salt stress

**Figure 6:**
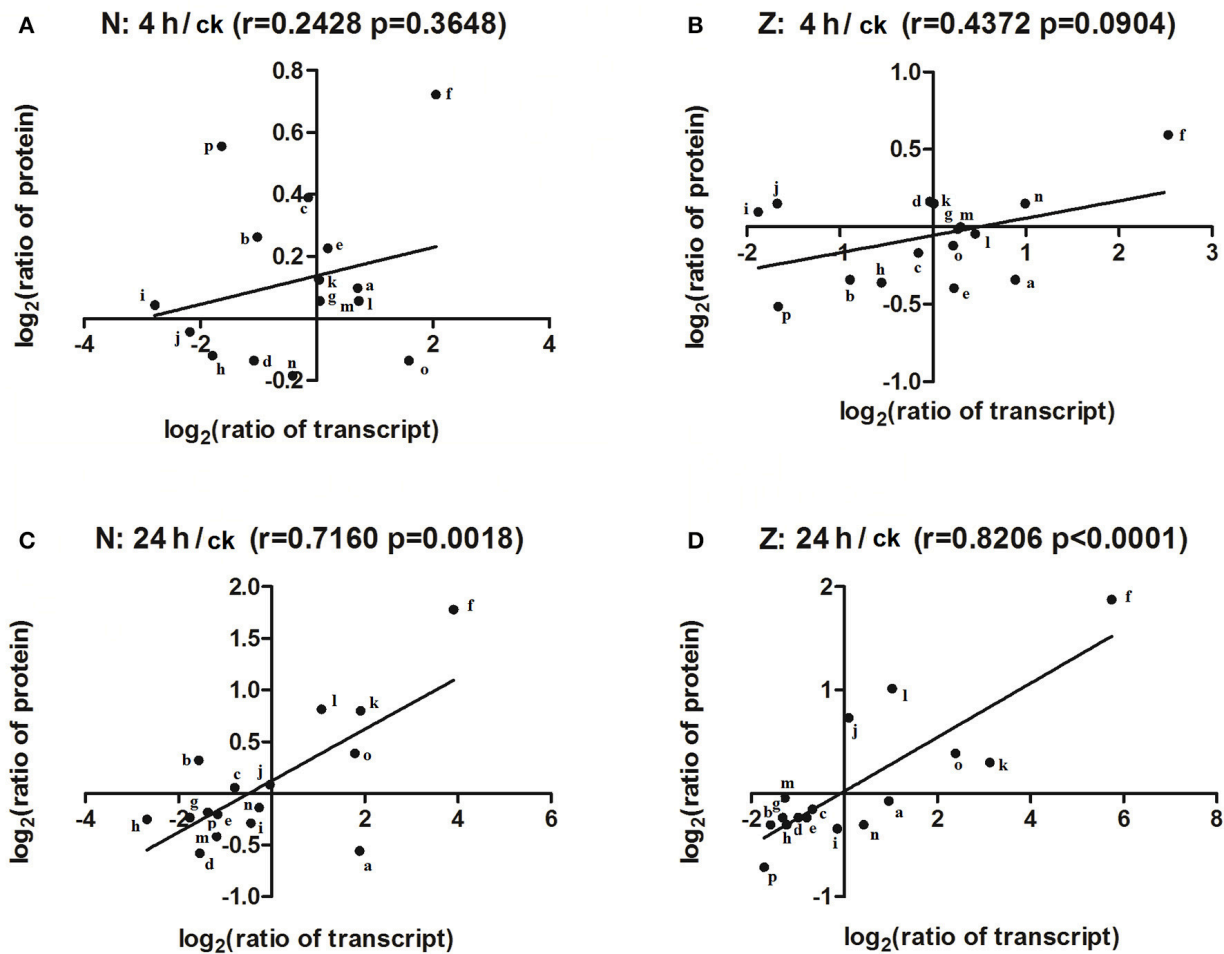
Correlation between changes in the abundance of DAPs and their matched unigenes. The correlation between the total DAPs and their matched transcript in the N4/Nck **(A)**, Z4/Zck **(B)**, N24/Nck **(C)**, and Z24/Zck **(D)** comparisons. The lines represent fitted straight trend lines from the data points. “r” represents the Pearson correlation coefficient. The letters “a”–“p” represent ATPase subunit d, Thylakoid lumenal 19-kDa protein, Ferredoxin–NADP reductase, RuBisCO large subunit-binding protein, Oxygen-evolving enhancer protein 3, Phosphoethanolamine N-methyltransferase, 30S ribosomal protein S17, Elongation factor Ts, 10 kDa chaperonin, Chaperonin CPN60-2, Probable nucleoredoxin 1, Glutathione S-transferase F9, Peptide methionine sulfoxide reductase B3, Pathogenesis-related protein STH-21, Peroxidase15, and unknown protein, respectively.

**Figure 7:**
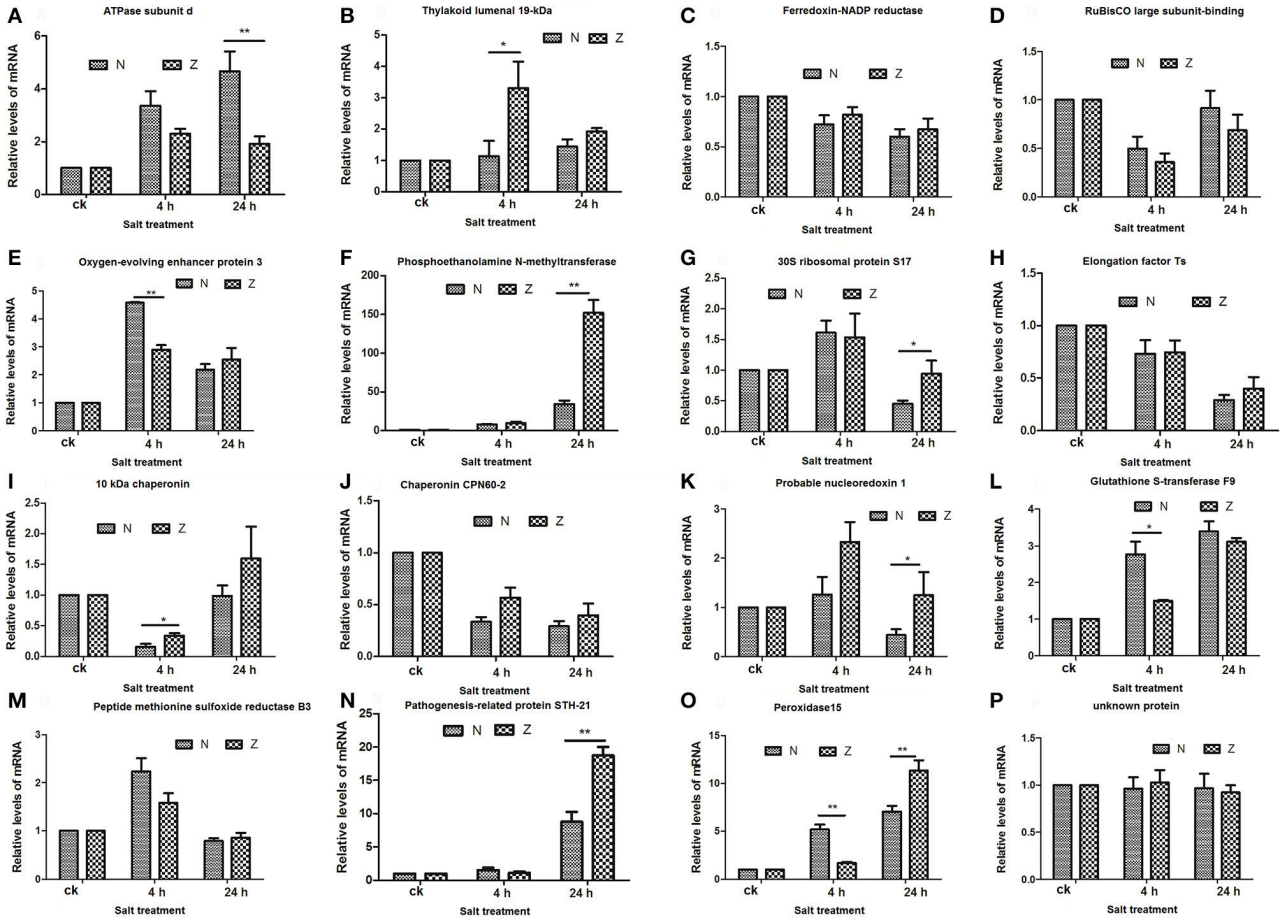
qRT-PCR results for the 16 matched DAPs in the proteome compared to the transcriptome. The relative mRNA expression levels of matched differentially abundant proteins including ATPase subunit d **(A)**, Thylakoid lumenal 19-kDa protein **(B)**, Ferredoxin-NADP reductase **(C)**, RuBisCO large subunit-binding protein **(D)**, Oxygen-evolving enhancer protein 3 **(E)**, Phosphoethanolamine N-methyltransferase **(F)**, 30S ribosomal protein S17 **(G)**, Elongation factor Ts **(H)**, 10 kDa chaperonin **(I)**, Chaperonin CPN60-2 **(J)**, Probable nucleoredoxin 1 **(K)**, Glutathione S-transferase F9 **(L)**, Peptide methionine sulfoxide reductase B3 **(M)**, Pathogenesis-related protein STH-21 **(N)**, Peroxidase15 **(O)**, and unknown protein **(P)**. Actin (Accession: AY305733) was used as the reference gene. The relative expression level of each gene was calculated using the formula the 2^−ΔΔCt^ (^*^P < 0.05, ^**^P < 0.01, Student's *t*-test). All the values shown are mean ± standard errors of three biological replicates, each of which has three technical replicate treatments.

To match proteins with unigene transcripts (Peng et al., 2014), we compared the differentially expressed genes and DAPs because the transcript and protein analyses shared the same technical samples and treatments. Sixteen of the 115 DAPs could be matched to transcripts (Supplementary Table S9), and the *r*-values between the total DAPs and the matched transcript fold-changes in the N4/Nck (Figure 6A), Z4/Zck (Figure 6B), N24/Nck (Figure 6C), and Z24/Zck (Figure 6D) comparisons were 0.2428, 0.4372, 0.7160, and 0.8206, respectively. For the 16 matched protein–unigene pairs, a greater level of overlap was obtained at 24 h than that at 4 h in both lines; Z had a higher *r*-value compared with N in both the treatment time points. Among these DAPs, phosphoethanolamine N-methyltransferase was the most highly induced protein, changing by 1.65-, 3.43-, 1.51-, and 3.67-fold in the N4/Nck, N24/Nck, Z4/Zck, and Z24/Zck comparisons, respectively. However, there were no obvious differences between N and Z. Besides, 16 matched proteins/unigenes were verified using qRT-PCR (Figure 7). The correlations between the qRT-PCR results and the protein abundances of these 16 matched proteins/genes in the N4/Nck, N24/Nck, Z4/Zck, and Z24/Zck comparisons were shown in Supplementary Figure S8.

## Discussion

### Salt stress produced differences in Na^+^ and K^+^ content between the 2 contrasting genotypes

Physiological analysis revealed that Z absorbed less Na^+^ and maintained lower ratios of Na^+^ to K^+^ in its leaves under salt stress for 24 h compared with N. Recently, Platten et al. (2013) evaluated 103 accessions from *Oryza sativa* and 12 accessions from *O. glaberrima* for salinity tolerance and found that the majority of tolerant accessions showed lower Na^+^ concentration in their leaves. This clearly indicates that low Na^+^ concentration in photosynthetically active leaves is an effective mechanism for salt tolerance. During salt stress, K^+^ is an essential ion for various physiological processes, particularly for protein synthesis and enzyme activation. Free radicals produced due to stress along with K^+^ deficiency is associated with programed cell death (Anschütz et al., 2014). High cytosolic Na^+^ concentrations and relatively low K^+^ concentrations can reduce plasma membrane H^+^-ATPase activity (Wakeel et al., 2011). In our data, lower abundances of the F0F1-type H^+^-ATP synthase gamma, delta, and epsilon subunits in Z compared with N were noticed after salt treatment for 4 h, and the mRNA expression levels of these proteins were also lower in Z. At this time point, salt stress resulted in no differences in Na^+^ content and Na^+^/K^+^ ratios but a lower K^+^ content in Z compared with N. It appears that the change in K^+^ concentration may be responsible for the H^+^-ATPase alteration at 4 h. At 24 h, only the H^+^-ATP synthase epsilon subunit was less abundant, with lower Na^+^ content and Na^+^/K^+^ ratios but higher K^+^ content in Z compared with N. However, the mRNA expression of the H^+^-ATP synthase epsilon subunit did not show significant differences between N and Z. Thus, our results indicated the presence of the specific and complex effects of Na^+^ and K^+^ on F0F1-type H^+^-ATP synthase in cotton. Vacuolar-type H^+^-ATPase (V-ATPase) plays an important role in inducing sodium sequestration into the central vacuole (Silva and Gerós, 2009; Peng et al., 2016). The V-ATPase subunit G was increased in abundance at 4 h but decreased at 24 h in N. However, it showed low abundance in Z. At 24 h, these 2 cotton genotypes secreted salt. However, the salt-tolerant genotype leaves could secrete more salt than the salt-sensitive genotype leaves (Peng et al., 2016). Further research is needed to clarify the relationship among salt secretion, K^+^, Na^+^, plasma membrane H^+^-ATPase, and V-ATPase in cotton.

### Salt stress affected chloroplast metabolism

Oxygen-evolving enhancer protein 3, which functions in oxygen evolution and maintains PSII stability, was less abundant in stressed leaves of Z and N after 24 h of salt treatment (Supplementary Table S7) and exhibited genotype specificity (with a ratio of 1.709 at 24 h). These changes protect reaction center proteins, and they are in accordance with previous studies on other plants in which Oxygen-evolving enhancer proteins responded to salinity and changed the activity of PSII (Zörb et al., 2009; Pang et al., 2010; Sobhanian et al., 2010; Bandehagh et al., 2011).

Upregulation of the PSI reaction center proteins may regulate the efficiency of electron transfer and transmembrane electrochemical gradients by affecting ATP synthesis and NADPH formation (Yang et al., 2013). We found that PSI P700 chlorophyll *a* apoprotein A2 (PSIP700), the primary electron donor of PSI, converting photonic excitation into a charge separation, was abundant at 4 h in the salt-tolerant genotype but low abundant in the salt-sensitive genotype (Supplementary Table S7). These results indicated that salt-tolerant cotton may have higher electron transfer efficiency than salt-sensitive cotton.

Few studies have been performed on the thylakoid lumenal 19 kDa protein, which is related to the PSII oxygen-evolving complex and calcium ion binding. It was highly abundant at 24 h in N but decreased in abundance in Z and showed no interaction in *A. thaliana*. Magnesium-chelatase subunit H, which was less abundant in both N and Z at 24 h, is a key component in both chlorophyll biosynthesis and plastid-to-nucleus signaling and is also an ABA receptor (Shen et al., 2006). The phytohormone ABA plays a vital role in plant development and response to environmental challenges such as salt accumulation and drought. Magnesium-chelatase subunit H had 14 interactions in the AtPID database, including 4 WRKY transcription factors.

### Salt stress-regulated ribosomal structure-related DAPs

Ribosomal proteins, as primary components of ribosomes, are mainly responsible for protein synthesis in cells. Previous studies have shown that *GmRPL37* markedly increases after soybeans are exposed to cold temperatures (Kim et al., 2004). Another ribosomal protein, AgRPS3aE, produced high salt tolerance in yeast (Liang et al., 2015). We also found 5 salt-responsive genotype-specific ribosomal proteins: 60S ribosomal protein L11-1 was high in abundance only in Z; 50S ribosomal protein L19-2 was abundant in Z but suppressed in N at 4 h; 40S ribosomal protein S17 and 50S ribosomal protein L16 were more abundant at 4 h; and 40S ribosomal protein S10 was less abundant at 4 h in Z but at 24 h in N. Among these, 40S ribosomal protein S10 showed 66 functional partners in the AtPID database.

The ATP binding-related protein DEAD-box known as ATP-dependent RNA helicase 3 was also involved in ribosomal structure and confers high salinity stress tolerance in plants (Amin et al., 2012; Sahoo et al., 2012). In our study, DEAD-box ATP-dependent RNA helicase 3 was low abundant in both the salt-tolerant and -sensitive genotypes under salt stress. However, no significant difference was noticed between N and Z.

### Salt stress-regulated phosphate metabolism and defense-related DAPs

Phosphoethanolamine N-methyltransferase 1 protein, an important enzyme in methylation metabolism, was highly abundant after 24 h of salt treatment in both N and Z. However, the abundance between N and Z did not show a significant difference. The silencing of phosphoethanolamine N-methyltransferase results in salt hypersensitivity in *Arabidopsis* (Mou et al., 2002; Hancock et al., 2005). Chang et al. (2014) found that the phosphoethanolamine N-methyltransferase gene was strongly induced by salt stress.

Fructose-1,6-bisphosphate aldolase, related to the pentose-phosphate shunt, was found in higher abundance in Z but was suppressed in N at 24 h and had 17 functional partners in the AtPID database. In *Arabidopsis*, the *Fructose-1,6-bisphosphate aldolase* knockout mutant exhibits sensitivity to salt stress (Moon et al., 2012). It was suggested that N induced reduction in glycolysis and carbon metabolism.

The transport and defense related protein thiamine thiazole synthase 2, involved in zinc ion binding and response to cold, was detected at low abundance in salt stress in N and Z. This protein has 16 interacting proteins, including the protein phosphorylated amino acid binding-related general regulatory factor GRF3.

Adenosylhomocysteinase, involved in copper ion binding and response to salt stress, was highly abundant upon exposure to salt stress, particularly in Z at 24 h. A total of 50 proteins interacted with adenosylhomocysteinase, including nucleoside diphosphate kinase 4 and mannose 6-phosphate reductase. However, in foxtail millet seedlings, adenosylhomocysteinase tended to be low abundant due to high salt stress (200 mM NaCl) (Veeranagamallaiaha et al., 2008).

### Salt stress-regulated golgi- and calcium-related DAPs

The Golgi apparatus plays a major role in the growth and division of plant cells due to its roles in protein glycosylation, protein sorting, and cell wall synthesis. In the current study, there were 27 (9.2%) Golgi-related salt-responsive DAPs. Many proteins that are located in the Golgi apparatus are induced by salt (Kang et al.,2008; Cubero et al., 2009). Protein N-glycosylation in the Golgi apparatus is an essential process in eukaryotic cells. Mutants that are defective in N-glycan maturation are more salt sensitive than the wild type (Kang et al., 2008). Fasciclin-like arabinogalactan protein 2, related to Golgi organization and calcium ion transport, showed low abundance after salt stress in Z and N. However, no difference was found between the 2 genotypes. Of 29 salt-responsive genotype-specific proteins (Supplementary Table S7), Golgi-related proteins included proteasome subunit alpha type-3, which was less abundant in N but showed high abundance in Z at 24 h, adenosylhomocysteinase, 60S ribosomal protein L11, ATP synthase subunit d, histone H4, and calmodulin-1/11/16.

The results of the current study showed that calreticulin was low abundant in only the salt-sensitive genotype and decreased in both the 4 and 24 h treatments. Another calcium lipid binding protein related to the Golgi apparatus was abundant in the salt-tolerant genotype after salt treatment for 4 h. Calmodulins are important mediators of Ca^2+^ signals and are found ubiquitously in all eukaryotic organisms. In our study, calmodulin-1/11/16, a Ca^2+^-binding protein, was related to Golgi vesicle transport and highly abundant after salt stress. After searching the AtPID database, 212 interacting proteins were identified.

Many studies have reported the role of calcium in the late secretory pathway; however, an emerging body of literature has implicated calcium in the regulation of protein trafficking through the Golgi apparatus. For instance, it is now appreciated that the Golgi apparatus is an inositol 1,4,5-trisphosphate-sensitive Ca^2+^ store and that the proteins involved in sequestering and releasing calcium are localized to the Golgi apparatus (Pizzo et al., 2010). After salt stress, these 2 cotton genotypes obviously secreted salt. However, in cotton and particularly in Z, salt secretion that is relevant to calcium and the Golgi apparatus requires more research.

## Conclusions

Our investigations provide some foundational information to reveal the complex mechanisms underlying salt tolerance in upland cotton. First, chloroplasts are the organelles responsible for photosynthesis, which is the primary process affected by salinity in cotton. In our study, approximately 54.9% of the salt-tolerant DAPs were related to chloroplast metabolism. Second, ATP-related proteins play an important role in the salt tolerance of cotton. In our data, 11.9% of the salt-tolerant DAPs in cotton were ATP-related. Among these, DEAD-box ATP-dependent RNA helicase 3, plasma membrane H^+^-ATPase, and V-type ATPase may play significant roles in salt stress. Third, salt stress apparently induced the changes in abundance of ribosomal proteins. Fourth, phosphate-related proteins contribute to the salt tolerance of cotton. After salt treatment, a phosphoethanolamine N-methyltransferase 1 protein that was related to secondary metabolite biosynthesis showed higher abundance in cotton at 24 h. Finally, it was noteworthy that Golgi- and calcium-related DAPs were induced under salt stress. The Golgi apparatus is a Ca^2+^ store, and many proteins involved in sequestering and releasing calcium are localized in the Golgi apparatus. Calcium played an important role in the late secretory pathway. To determine if salt secretion in cotton is associated with calcium and the Golgi apparatus requires more research.

## Availability of data

All of the raw mass spectra files in LC-MS/MS have been deposited into the publicly accessible database PeptideAtlas and now are available using dataset Identifier PASS00856 (http://www.peptideatlas.org/PASS/PASS00856).

## Author contributions

XD received grant support. XD and WG designed the experiment. FX grew the cotton seedlings and performed the physiological experiments. WG and FX analyzed the physiological and proteome results and prepared the manuscript. JS and ZP participated in the management of cotton cultivation. SH, ZP and XD revised the manuscript. All authors reviewed and approved the final manuscript.

### Conflict of interest statement

The authors declare that the research was conducted in the absence of any commercial or financial relationships that could be construed as a potential conflict of interest.

## References

[B1] Amin M. Hossain A. Ferdousi A. Rahman M. Tuteja N. (2012). Over-expression of a dead-box helicase, PDH45, confers both seedling and reproductive stage salinity tolerance to rice (*Oryza sativa* L.). Mol. Breed. 30, 345–354. 10.1007/s11032-011-9625-3

[B2] Anschütz U. Becker D. Shabala S. (2014). Going beyond nutrition: regulation of potassium homoeostasis as a common denominator of plant adaptive responses to environment. J. Plant Physiol. 171, 670–687. 10.1016/j.jplph.2014.01.009 24635902

[B3] Ashraf M. (2002). Salt tolerance of cotton: some new advances. CRC Crit. Rev. Plant Sci. 21, 1–30. 10.1016/S0735-2689(02)80036-3

[B4] Bandehagh A. Salekdeh G. H. Toorchi M. Mohammadi A. Komatsu S. (2011). Comparative proteomic analysis of canola leaves under salinity stress. Proteomics. 11, 1965–1975. 10.1002/pmic.201000564 21480525

[B5] Basal H. Hemphill J. K. Smith C. W. (2006). Shoot and root characteristics of converted race stocks accessions of upland cotton (*Gossypium hirsutum* L.) grown under salt stress conditions. Am. J. Plant Physiol. 1, 99–106. 10.3923/ajpp.2006.99.106

[B6] Bose J. Rodrigo-Moreno A. Lai D. Xie Y. Shen W. Shabala S. (2014). Rapid regulation of the plasma membrane H^+^-ATPase activity is essential to salinity tolerance in two halophyte species, *Atriplex lentiformis* and *Chenopodium quinoa*. Ann. Bot. 115, 481–494. 10.1093/aob/mcu219 25471095 PMC4332608

[B7] Cao W. H. Liu J. He X. J. Mu R. L. Zhou H. L. Chen S. Y.. (2007). Modulation of ethylene responses affects plant salt-stress responses. Plant Physiol. 143, 707–719. 10.1104/pp.106.094292 17189334 PMC1803741

[B8] Chang D. Zhang X. Zhang F. (2014). Cloning and expression analysis of hcpeamt gene from *Halostachys caspica*. Acta Botanica Boreali-Occidentalia Sinica. 34, 1522–1528. 10.7606/j.issn.1000-4025.2014.08.1522

[B9] Cubero B. Nakagawa Y. Jiang X. Y. Miura K. J. Li F. Raghothama K. G.. (2009). Raghothama KG, The phosphate transporter PHT4;6 is a determinant of salt tolerance that is localized to the golgi apparatus. Mol. Plant. 2, 535–552. 10.1093/mp/ssp013 19825636

[B10] Cui Y. Fan B. Wang D. Wang J. Wang S. Ye W. (2012). Analysis of differential proteins of cotton leaves under salt stress. Mol. Plant Breed. 10, 48–54. 10.3969/mpb.010.000048

[B11] Diray-Arce J. Clement M. Gul B. Khan M. A. Nielsen B. L. (2015). Transcriptome assembly, profiling and differential gene expression analysis of the halophyte *Suaeda fruticosa* provides insights into salt tolerance. BMC Genomics. 6, 353. 10.1186/s12864-015-1553-x 25943316 PMC4422317

[B12] Du S. J. Dong C. J. Zhang B. Lai T. F. Du X. M. Liu J. Y. (2013). Comparative proteomic analysis reveals differentially expressed proteins correlated with fuzz fiber initiation in diploid cotton (*Gossypium arboreum* L.). J. Proteomics. 82, 113–129. 10.1016/j.jprot.2013.02.020 23474080

[B13] Du X. Sun J. Zhou Z. Jia Y. Pan Z. He S. (2012). Current situation and the future in collection, preservation, evaluation and utilization of cotton germplasm in China. J. Plant Gen. Res. 13, 163–168. 10.13430/j.cnki.jpgr.2012.02.003

[B14] El Rabey H. A. Al-Malki A. L. Abulnaja K. O. (2016). Proteome analysis of date palm (*Phoenix dactylifera* L.) under severe drought and salt stress. Int. J. Genomics. 2016:7840759. 10.1155/2016/7840759 27840818 PMC5093262

[B15] Gao S. Q. Chen M. Xia L. Q. Xiu H. J. Xu Z. S. Li L. C.. (2009). A cotton (*Gossypium hirsutum*) DRE-binding transcription factor gene, GhDREB, confers enhanced tolerance to drought, high salt, and freezing stresses in transgenic wheat. Plant Cell Rep. 28, 301–311. 10.1007/s00299-008-0623-9 19005655

[B16] Ghars M. A. Parre E. Debez A. Bordenave M. Richard L. Leportc L.. (2008). Comparative salt tolerance analysis between *Arabidopsis thaliana* and *Thellungiella halophila*, with special emphasis on K^+^/Na^+^ selectivity and proline accumulation. J. Plant Physiol. 165, 588–599. 10.1016/j.jplph.2007.05.014 17723252

[B17] Guo G. Ge P. Ma C. Li X. Lv D. Wang S.. (2012). Comparative proteomic analysis of salt response proteins in seedling roots of two wheat varieties. J. Proteomics. 75, 1867–1885. 10.1016/j.jprot.2011.12.032 22245046

[B18] Hancock J. T. Henson D. Nyirenda M. Desikan R. Harrison J. Lewis M.. (2005). Proteomic identification of glyceraldehyde 3-phosphate dehydrogenase as an inhibitory target of hydrogen peroxide in Arabidopsis. Plant Physiol. Biochem. 43, 828–835. 10.1016/j.plaphy.2005.07.012 16289945

[B19] Hu G. Koh J. Yoo M. J. Grupp K. Chen S. Wendel J. F. (2013). Proteomic profiling of developing cotton fibers from wild and domesticated *Gossypium barbadense*. New Phytol. 200, 570–582. 10.1111/nph.12381 23795774

[B20] Kang J. S. Frank J. Kang C. H. Kajiura H. Vikram M. Ueda A. (2008). Salt tolerance of *Arabidopsis thaliana* requires maturation of N-glycosylated proteins in the golgi apparatus. Proc. Natl. Acad. Sci. U.S.A. 10, 5933–5938. 10.1073/pnas.0800237105 PMC2311335 18408158

[B21] Katam R. Sakata K. Suravajhala P. Pechan T. Kambiranda D. M. Naik K. S.. (2016). Comparative leaf proteomics of drought-tolerant and -susceptible peanut in response to water stress. J. Proteom. 143, 209–226. 10.1016/j.jprot.2016.05.031 27282920

[B22] Katam R. Basha S. M. Suravajhala P. Pechan T. (2010). Analysis of peanut leaf proteome. J. Proteome Res. 9, 2236–2254. 10.1021/pr901009n 20345176

[B23] Kim K. Y. Park S. W. Chung Y. S. Chung C. H. Kim J. I. Lee J. H. (2004). Molecular cloning of low-temperature-inducible ribosomal proteins from soybean. J. Exp. Bot. 55, 1153–1155. 10.1093/jxb/erh125 15020631

[B24] Kumar S. A. Kumari P. H. Jawahar G. Prashanth S. Suravajhala P. Katam R. (2016). Beyond just being foot soldiers-osmotin like protein (OLP) and chitinase (Chi11) genes act as sentinels to confront salt, drought, and fungal stress tolerance in tomato. Environ. Exp. Bot. 132, 53–65. 10.1016/j.envexpbot.2016.08.007

[B25] Kumar S. A. Kumari P. H. Kumar G. S. Mohanalatha C. Kishor P. B. K. (2015). Osmotin: a plant sentinel and a possible agonist of mammalian adiponectin. Front. Plant Sci. 6:163 10.3389/fpls.2015.00163 25852715 PMC4360817

[B26] Kumari P. H. Kumar S. A. Sivan P. Katam R. Suravajhala P. Rao K. S.. (2017). Overexpression of a plasma membrane bound Na^+^/H^+^ antiporter-like protein (SbNHXLP) confers salt tolerance and improves fruit yield in tomato by maintaining ion homeostasis. Front. Plant Sci. 7:2027. 10.3389/fpls.2016.02027 28111589 PMC5216050

[B27] Liang X. Liu Y. Xie L. Liu X. Wei Y. Zhou X.. (2015). A ribosomal protein AgRPS3aE from halophilic *Aspergillus glaucus* confers salt tolerance in heterologous organisms. Int. J. Mol. Sci. 16, 3058–3070. 10.3390/ijms16023058 25642759 PMC4346880

[B28] Maier T. Güell M. Serrano L. (2009). Correlation of mRNA and protein in complex biological samples. FEBS Lett. 583, 3966–3973. 10.1016/j.febslet.2009.10.036 19850042

[B29] Meng C. Cai C. Zhang T. Guo W. (2009). Characterization of six novel NAC genes and their responses to abiotic stresses in *Gossypium hirsutum* L. Plant Sci. 17, 352–359. 10.1016/j.plantsci.2008.12.003

[B30] Moon S. J. Shin D. Kim B. G. Byun M. O. (2012). Putative fructose-1,6- bisphosphate aldolase 1 (AtFBA1) affects stress tolerance in yeast and Arabidopsis. J. Plant Biotechnol. 39, 106–113. 10.5010/JPB.2012.39.2.106

[B31] Mou Z. Wang X. Fu Z. Dai Y. Han C. Ouyang J. (2002). Silencing of Phosphoethanolamine N-methyltransferase results in temperature-sensitive male sterility and salt hypersensitivity in Arabidopsis. Plant Cell. 14, 2031–2043. 10.1105/tpc.001701 12215503 PMC150753

[B32] Munns R. James R. A. Läuchli A. (2006). Approaches to increasing the salt tolerance of wheat and other cereals. J. Exp. Bot. 57, 1025–1043. 10.1093/jxb/erj100 16510517

[B33] Munns R. Tester M. (2008). Mechanisms of salinity tolerance. Annu. Rev. Plant Biol. 59, 651–681. 10.1146/annurev.arplant.59.032607.092911 18444910

[B34] Pang Q. Chen S. Dai S. Chen Y. Wang Y. Yan X. (2010). Comparative proteomics of salt tolerance in *Arabidopsis thaliana* and *Thellungiella halophila*. J. Proteome Res. 9, 2584–2599. 10.1021/pr100034f 20377188

[B35] Peng Z. He S. Gong W. Sun J. Pan Z. Xu F.. (2014). Comprehensive analysis of differentially expressed genes and transcriptional regulation induced by salt stress in two contrasting cotton genotypes. BMC Genomics 15:760. 10.1186/1471-2164-15-760 25189468 PMC4169805

[B36] Peng Z. He S. Sun J. Pan Z. Gong W. Lu Y.. (2016). Na^+^ compartmentalization related to salinity stress tolerance in upland cotton (*Gossypium hirsutum*) seedlings. Sci. Rep. 6:34548. 10.1038/srep34548 27698468 PMC5048304

[B37] Pizzo P. Lissandron V. Pozzan T. (2010). The trans-golgi compartment: a new distinct intracellular Ca^2+^ store. J. Commun. Integr. Biol. 3, 462–464. 10.4161/cib.3.5.12473 PMC2974081 21057641

[B38] Platten J. D. Egdane J. A. Ismail A. M. (2013). Salinity tolerance, Na^+^ exclusion and allele mining of HKT1;5 in *Oryza sativa* and *O. glaberrima*: many sources, many genes, one mechanism? BMC Plant Biol. 13:32. 10.1186/1471-2229-13-32 23445750 PMC3599985

[B39] Qin J. Gu F. Liu D. Yin C. Zhao S. Chen H.. (2013). Proteomic analysis of elite soybean Jidou17 and its parents using iTRAQ-based quantitative approaches. Proteome Sci. 11:12. 10.1186/1477-5956-11-12 23531047 PMC3622570

[B40] Sahoo R. K. Gill S. S. Tuteja N. (2012). Pea DNA helicase 45 promotes salinity stress tolerance in IR64 rice with improved yield. Plant Sign. Behav. 7, 1042–1046. 10.4161/psb.20915 22827940 PMC3474676

[B41] Schmittgen T. D. Livak K. J. (2008). Analyzing real-timePCR data by the comparative CT method. Nat. Protoc. 3, 1101–1108. 10.1038/nprot.2008.73 18546601

[B42] Shabala S. Cuin A. T. (2007). Potassium transport and plant salt tolerance. Physiol. Plant. 133, 651–669. 10.1111/j.1399-3054.2007.01008.x 18724408

[B43] Shen Y. Y. Wang X. F. Wu F. Q. Du S. Y. Cao Z. Shang Y.. (2006). The Mg-chelatase H subunit is an abscisic acid receptor. Nature 443, 823–826. 10.1038/nature05176 17051210

[B44] Silva P. Gerós H. (2009). Regulation by salt of vacuolar H^+^-ATPase and H^+^-pyrophosphatase activities and Na^+^/H^+^ exchange. Plant Signal. Behav. 4, 718–726. 10.4161/psb.4.8.9236 19820346 PMC2801382

[B45] Silveira J. A. G. Carvalho F. E. L. (2016). Proteomics, photosynthesis and salt resistance in crops: an integrative view. J. Proteomics 143, 24–35. 10.1016/j.jprot.2016.03.013 26957143

[B46] Sobhanian H. Motamed N. Jazii F. R. Nakamura T. Komatsu S. (2010). Salt stress induced differential proteome and metabolome response in the shoots of *Aeluropus lagopoides* (Poaceae), a halophyte C4 plant. J. Proteome Res. 9, 2882–2997. 10.1021/pr900974k 20397718

[B47] Tang X. Mu X. Shao H. Wang H. Brestic M. (2015). Global plant-responding mechanisms to salt stress: physiological and molecular levels and implications in biotechnology. Crit. Rev. Biotechnol. 35, 425–437. 10.3109/07388551.2014.889080 24738851

[B48] Venisse J. S. Gullner G. Brisset M. N. (2001). Evidence for the involvement of an oxidative stress in the initiation of infection of pear by *Erwinia amylovora*. Plant Physiol. 125, 2164–2172. 10.1104/pp.125.4.2164 11299395 PMC88871

[B49] Veeranagamallaiaha G. Jyothsnakumaria G. Thippeswamya M. Chandra Obul Reddya P. Surabhia G. K. Sriranganayakulua G. (2008). Proteomic analysis of salt stress responses in foxtail millet (*Setaria italica* L. cv. Prasad) seedlings. Plant Sci. 175, 631–641. 10.1016/j.plantsci.2008.06.017

[B50] Wakeel A. Sümer A. Hanstein S. Yan F. Schubert S. (2011). *In vitro* effect of different Na^+^/K^+^ ratios on plasma membrane H^+^ -ATPase activity in maize and sugar beet shoot. Plant. Physiol. Biochem. 49, 341–345. 10.1016/j.plaphy.2011.01.006 21282062

[B51] Wang L. Pan D. Li J. Tan F. Hoffmann-Benning S. Liang W.. (2015). Proteomic analysis of changes in the *Kandelia candel* chloroplast proteins reveals pathways associated with salt tolerance. Plant Sci. 231, 159–172. 10.1016/j.plantsci.2014.11.013 25576001

[B52] Wong C. E. Li Y. Labbe A. Guevara D. Nuin P. Whitty B.. (2006). Transcriptional profiling implicates novel interactions between abiotic stress and hormonal responses in Thellungiella, a close relative of Arabidopsis. Plant Physiol. 140, 1437–1450. 10.1104/pp.105.070508 16500996 PMC1435811

[B53] Yang L. Zhang Y. Zhu N. Koh J. Ma C. Pan Y.. (2013). Proteomic analysis of salt tolerance in sugar beet monosomic addition line M14. J. Proteome Res. 12, 4931–4950. 10.1021/pr400177m 23799291

[B54] Yao D. Zhang X. Zhao X. Liu C. Wang C. Zhang Z.. (2011). Transcriptome analysis reveals salt-stress-regulated biological processes and key pathways in roots of cotton (*Gossypium hirsutum* L.). Genomics 98, 47–55. 10.1016/j.ygeno.2011.04.007 21569837

[B55] Zhang H. Han B. Wang T. Chen S. Li H. Zhang Y.. (2012). Mechanisms of plant salt response: insights from proteomics. J. Proteome Res. 11, 49–67. 10.1021/pr200861w 22017755

[B56] Zhang X. Yao D. Wang Q. Xu W. Wei Q. Wang C.. (2013). mRNA-seq analysis of the *Gossypium arboreum* transcriptome reveals tissue selective signaling in response to water stress during seedling stage. PLoS ONE 8:e54762. 10.1371/journal.pone.0054762 23382961 PMC3557298

[B57] Zhang X. Zhen J. Li Z. Kang D. Yang Y. Kong J. (2011). Expression profile of early responsive genes under salt stress in upland cotton (*Gossypium hirsutum* L.). Plant Mol. Biol. Rep. 29, 626–637. 10.1007/s11105-010-0269-y

[B58] Zheng M. Meng Y. Yang C. Zhou Z. Wang Y. Chen B. (2014). Protein expression changes during cotton fiber elongation in response to drought stress and recovery. Proteomics 14, 1776–1795. 10.1002/pmic.201300123 24889071

[B59] Zhou J. Wang X. Jiao Y. Qin Y. Liu X. He K.. (2007). Global genome expression analysis of rice in response to drought and high-salinity stresses in shoot, flag leaf, and panicle. Plant Mol. Biol. 63, 591–608. 10.1007/s11103-006-9111-1 17225073 PMC1805039

[B60] Zörb C. Herbst R. Forreiter C. Schubert S. (2009). Short-term effects of salt exposure on the maize chloroplast protein pattern. Proteomics 9, 4209–4220. 10.1002/pmic.200800791 19688749

