# Supplementary material for: iTRAQ-Based Comparative Proteomic Analysis of Seedling Leaves of Two Upland Cotton Genotypes Differing in Salt Tolerance
Source: Front Plant Sci. 2017 Dec 13;8:2113. doi: 10.3389/fpls.2017.02113 (PMC5733471; doi:10.3389/fpls.2017.02113)
Supplement: Supplementary file 10 [file Image1.PDF]

Title Page

iTRAQ-based comparative proteomic analysis of seedling leaves of two upland cotton genotypes differing in salt tolerance

Wenfang Gong[†], Feifei Xu[†], Junling Sun, Zhen Peng, Shoupu He, Zhaoe Pan, Xiongming Du[*]

† This author contributed equally to this work
* To whom correspondence should be addressed

1. Wenfang Gong
Address: State Key Laboratory of Cotton Biology, Institute of Cotton Research, Chinese Academy of Agricultural Sciences, Anyang, Henan 455000, China.
Email: gongwenfang@caas.cn

2. Feifei Xu
Address: State Key Laboratory of Cotton Biology, Institute of Cotton Research, Chinese Academy of Agricultural Sciences, Anyang, Henan 455000, China.
Email: xff518@126.com

3. Junling Sun
Address: State Key Laboratory of Cotton Biology, Institute of Cotton Research, Chinese Academy of Agricultural Sciences, Anyang, Henan 455000, China
Email: sunjl000@163.com

4. Zhen Peng
Address: State Key Laboratory of Cotton Biology, Institute of Cotton Research, Chinese Academy of Agricultural Sciences, Anyang, Henan 455000, China
Email: cripengzhen09@126.com

5. Shoupu He
Address: State Key Laboratory of Cotton Biology, Institute of Cotton Research, Chinese Academy of Agricultural Sciences, Anyang, Henan 455000, China
Email: zephyr0911@126.com

6. Zhaoe Pan
Address: State Key Laboratory of Cotton Biology, Institute of Cotton Research, Chinese Academy of Agricultural Sciences, Anyang, Henan 455000, China
Email: panze@cricaas.com.cn

7. Xiongming Du (*Corresponding author)
Address: State Key Laboratory of Cotton Biology, Institute of Cotton Research, Chinese Academy of Agricultural Sciences, Anyang, Henan 455000, China
Telephone: 0086-372-2562252,
Fax: 0086-372-2562256
Email: dujeffrey8848@hotmail.com

Supplementary Figure S1. An SDS-PAGE (12% gels) was performed to verify the protein quality and concentration. A total of 30 μg of the protein sample was added to the gel. Three biological replicates were performed.

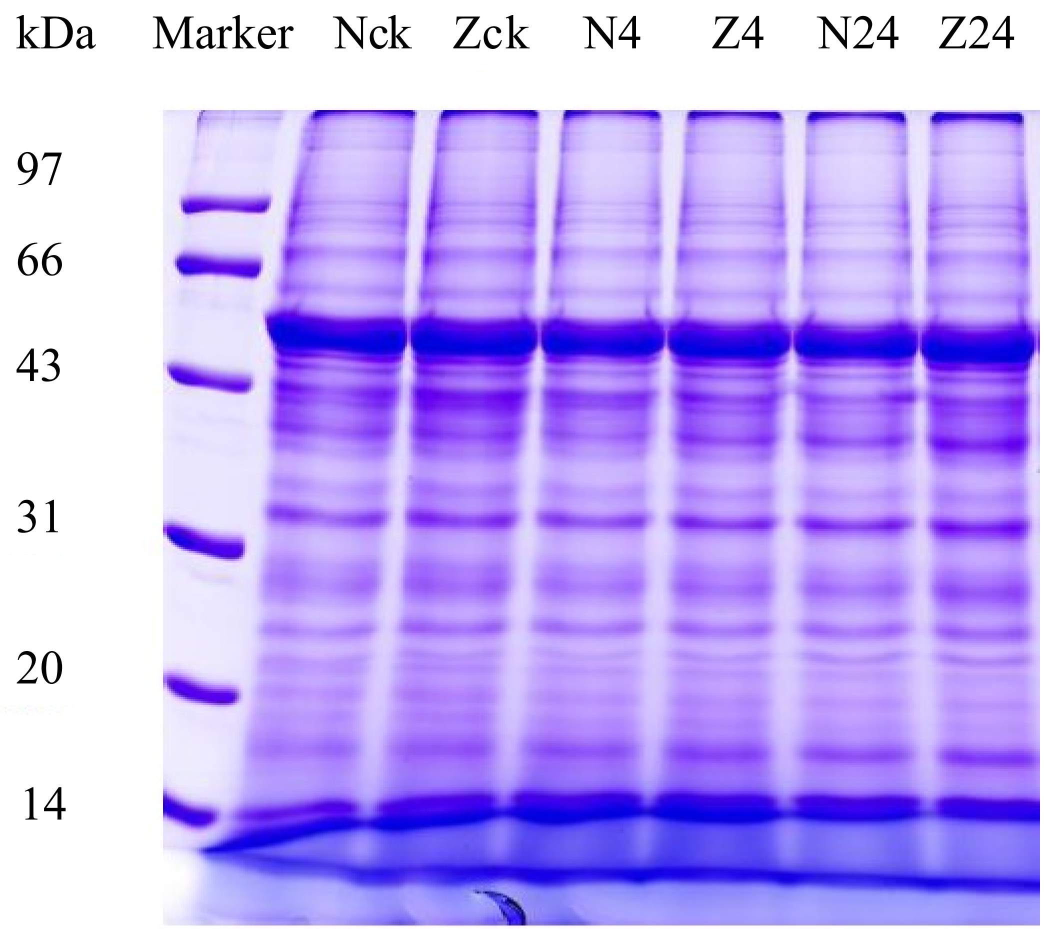

Supplementary Figure S2. The GO analyses of total identified proteins of Nan Dan Ba Di Da Hua and Earlistaple 7 subjected to 200 mM

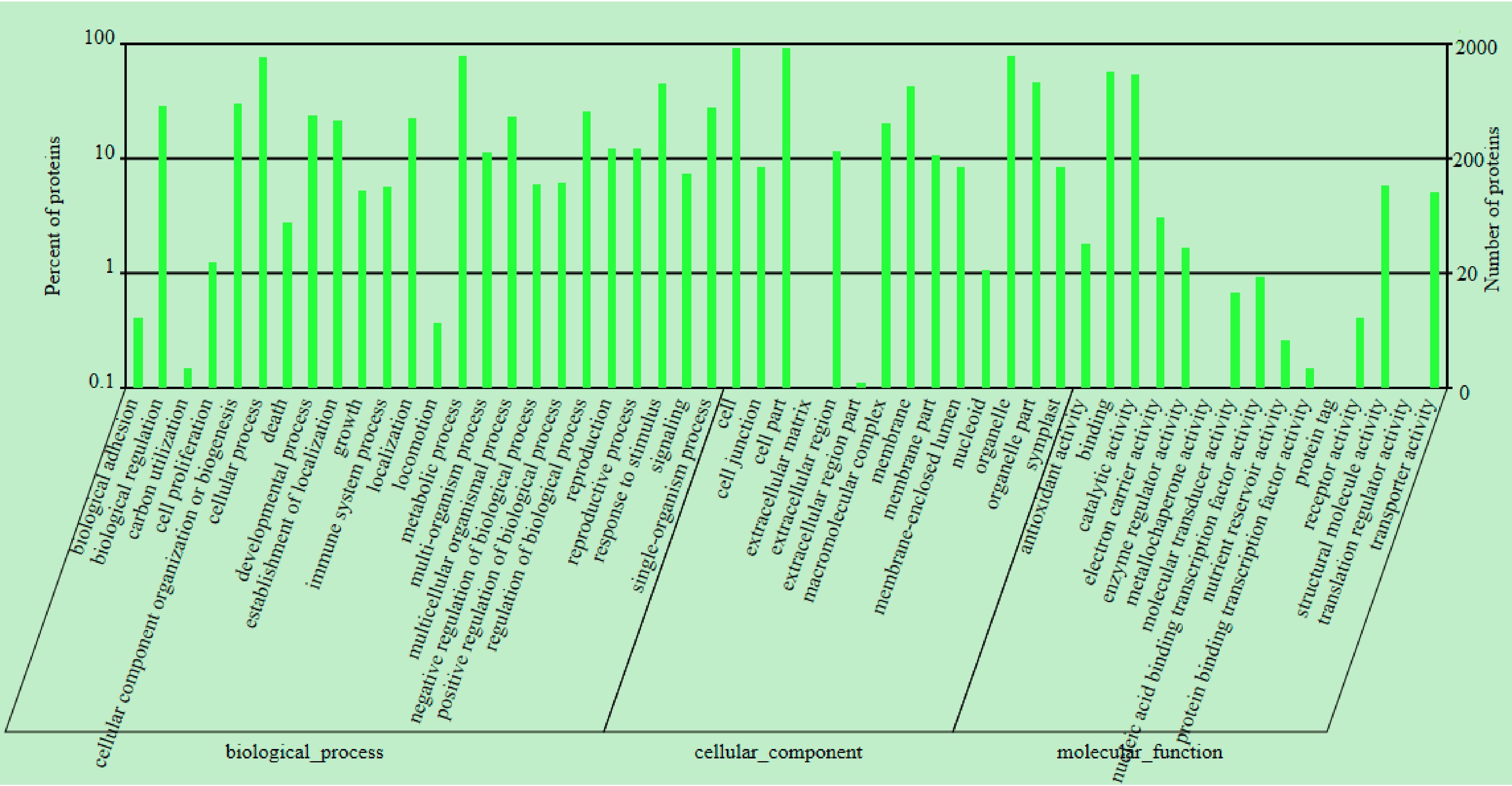

Supplementary Figure S3. The GO and KEGG analyses of differentially abundant proteins of Nan Dan Ba Di Da Hua and Earlistaple 7 subjected to 200 mM NaCl. GO categories of biological process (A), cellular component (B) and molecular function (C) with the top 20 enriched terms, and (D) KEGG pathway based on P-values. All the GO terms and KEGG pathway were significantly enriched based on P-values <0.05

A

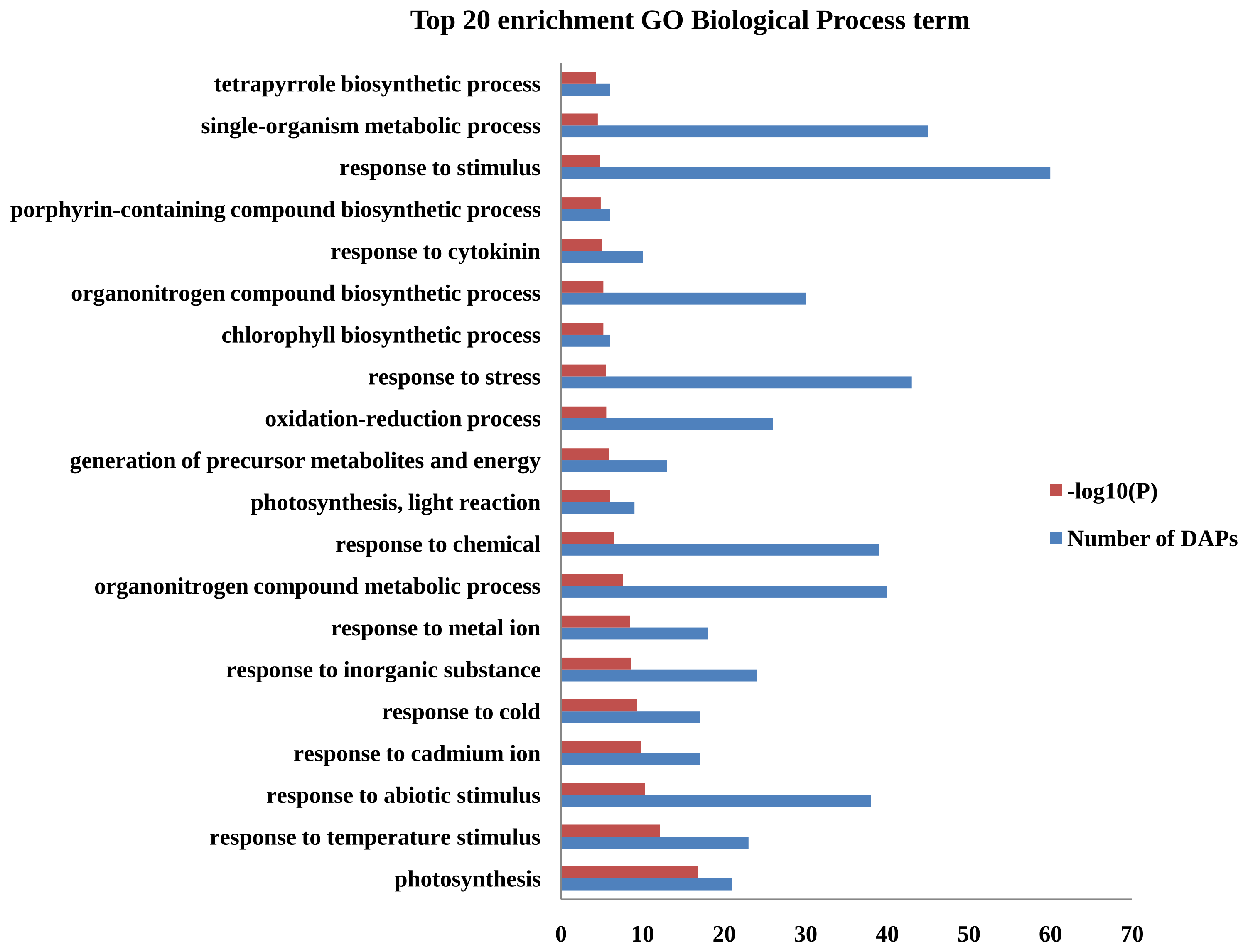
Top 20 enrichment GO Biological Process term
tetrapyrrole biosynthetic process
single-organism metabolic process
response to stimulus
porphyrin-containing compound biosynthetic process
response to cytokinin
organonitrogen compound biosynthetic process
chlorophyll biosynthetic process
response to stress
oxidation-reduction process
generation of precursor metabolites and energy
photosynthesis, light reaction
response to chemical
organonitrogen compound metabolic process
response to metal ion
response to inorganic substance
response to cold
response to cadmium ion
response to abiotic stimulus
response to temperature stimulus
photosynthesis
0
10
20
30
40
50
60
70
-log10(P)
Number of DAPs

B

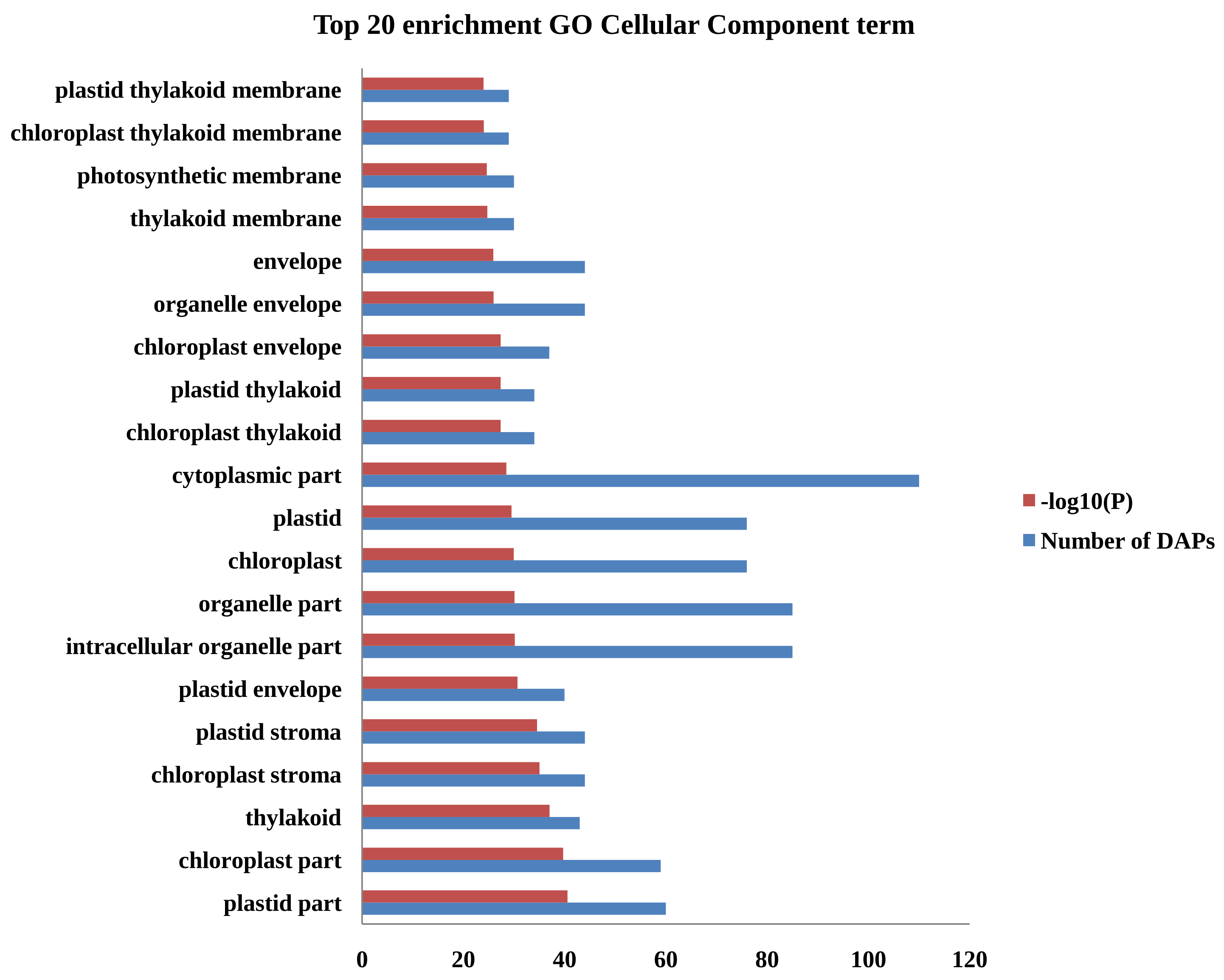
Top 20 enrichment GO Cellular Component term
plastid thylakoid membrane
chloroplast thylakoid membrane
photosynthetic membrane
thylakoid membrane
envelope
organelle envelope
chloroplast envelope
plastid thylakoid
chloroplast thylakoid
cytoplasmic part
plastid
chloroplast
organelle part
intracellular organelle part
plastid envelope
plastid stroma
chloroplast stroma
thylakoid
chloroplast part
plastid part
0
20
40
60
80
100
120
-log10(P)
Number of DAPs

C

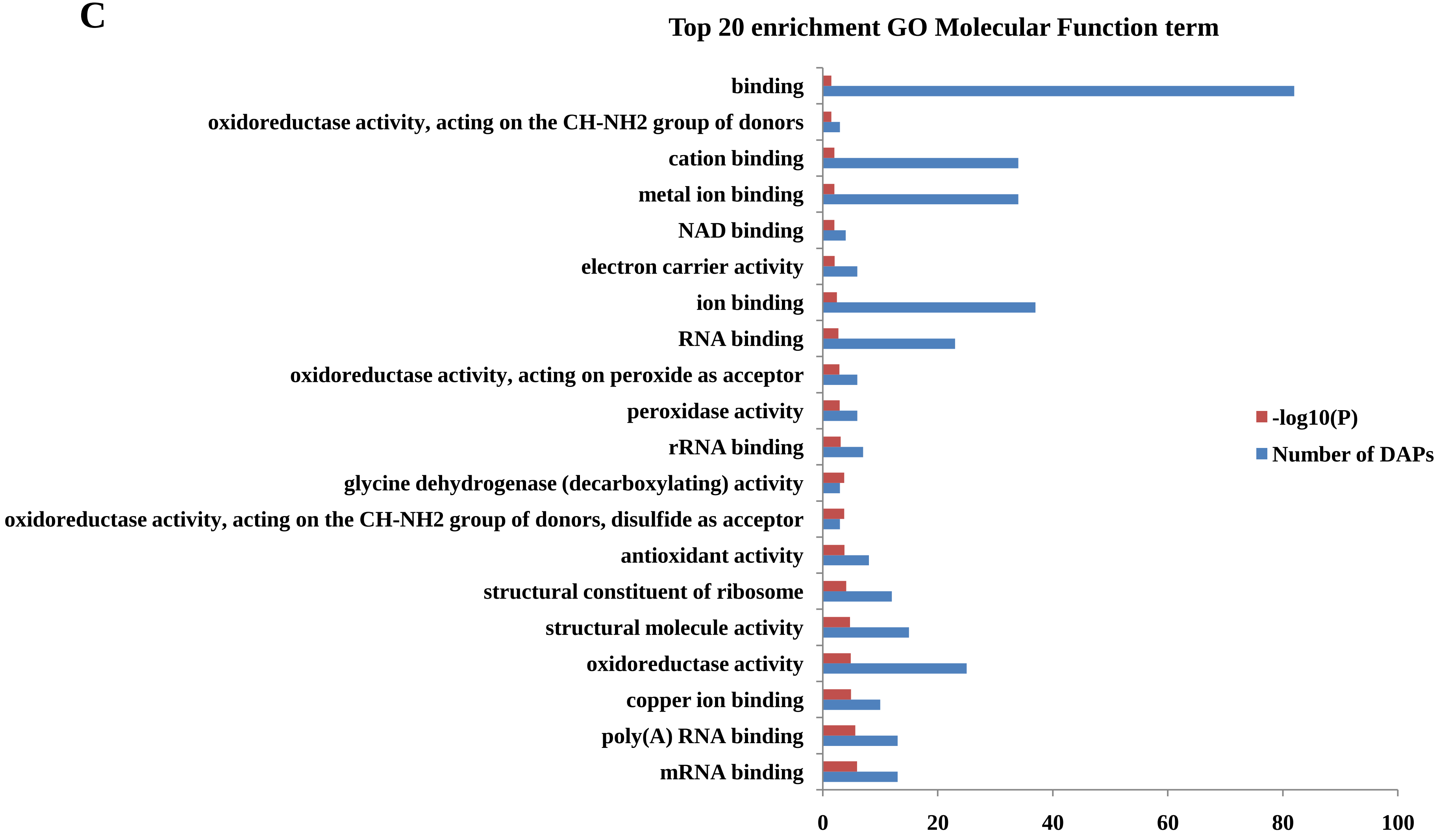
Top 20 enrichment GO Molecular Function term
binding
oxidoreductase activity, acting on the CH-NH2 group of donors
cation binding
metal ion binding
NAD binding
electron carrier activity
ion binding
RNA binding
oxidoreductase activity, acting on peroxide as acceptor
peroxidase activity
rRNA binding
glycine dehydrogenase (decarboxylating) activity
oxidoreductase activity, acting on the CH-NH2 group of donors, disulfide as acceptor
antioxidant activity
structural constituent of ribosome
structural molecule activity
oxidoreductase activity
copper ion binding
poly(A) RNA binding
mRNA binding
-log10(P)
Number of DAPs
0
20
40
60
80
100

D

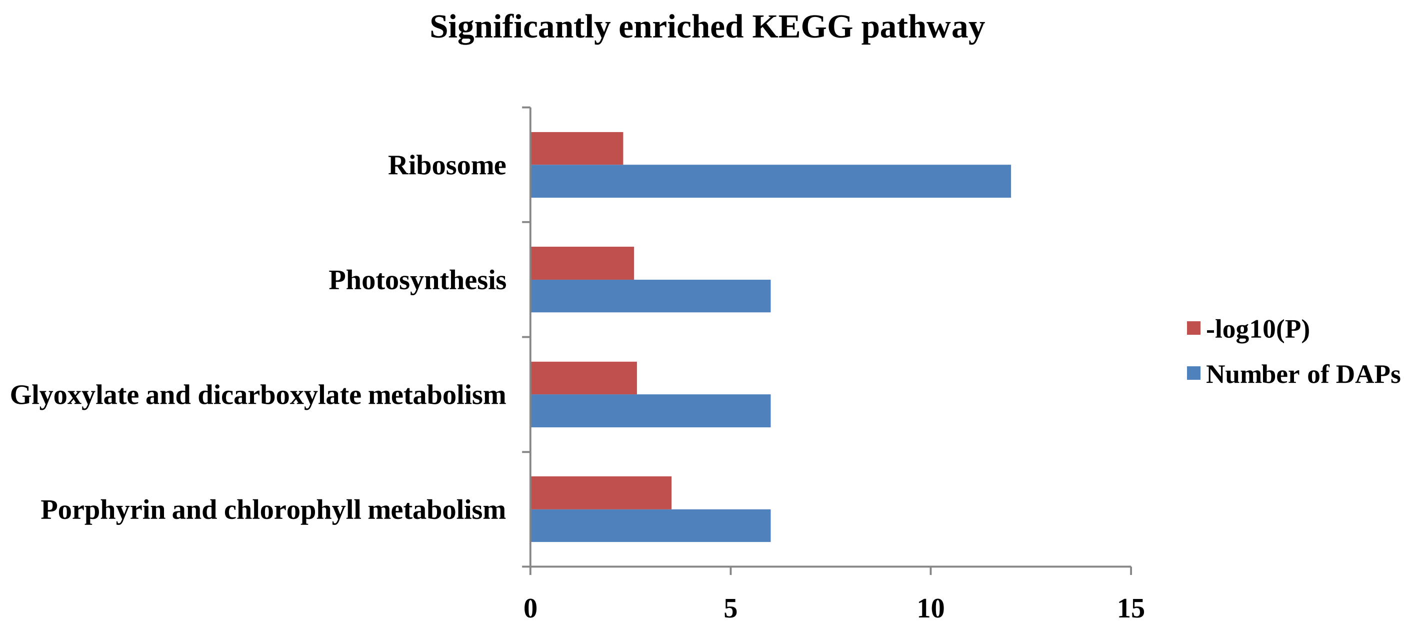

Significantly enriched KEGG pathway
Ribosome
Photosynthesis
Glyoxylate and dicarboxylate metabolism
Porphyrin and chlorophyll metabolism
0
5
10
15
-log10(P)
Number of DAPs

Supplementary Figure S4. The characters of salt-responsive (A) and genotype-specific (B) DAPs in Nan Dan Ba Di Da Hua (N) and Earlistaple 7 (Z) subjected to 200 mM NaCl.

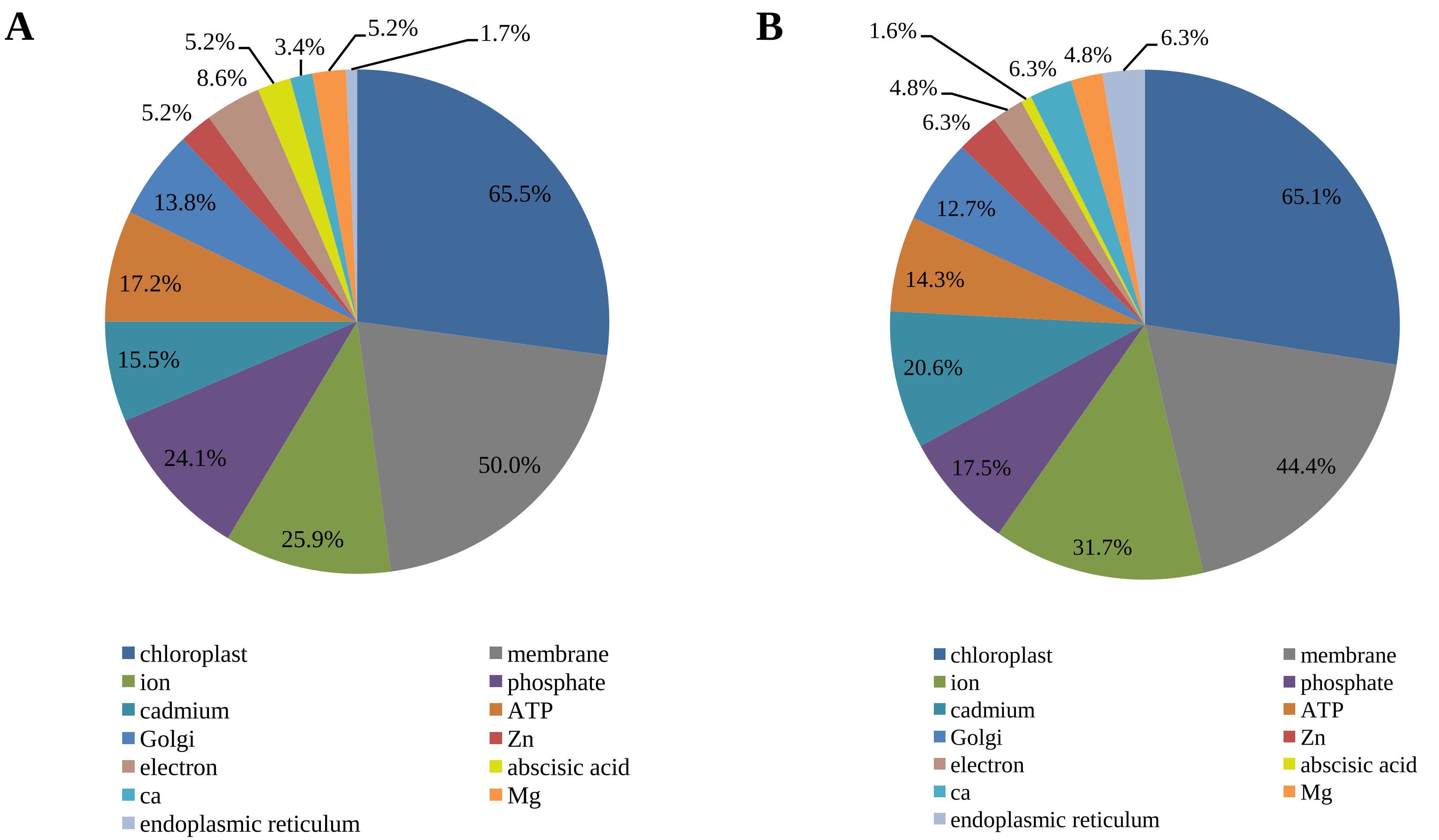

Supplementary Figure S5. Group analyses of salt-responsive proteins significantly changed in abundance under salt treatment. Six groups were generated to classify differentially abundant proteins in the 4 comparing sets N4/Nck, N24/Nck, Z4/Zck and Z24/Zck.

Supplementary Figure S5

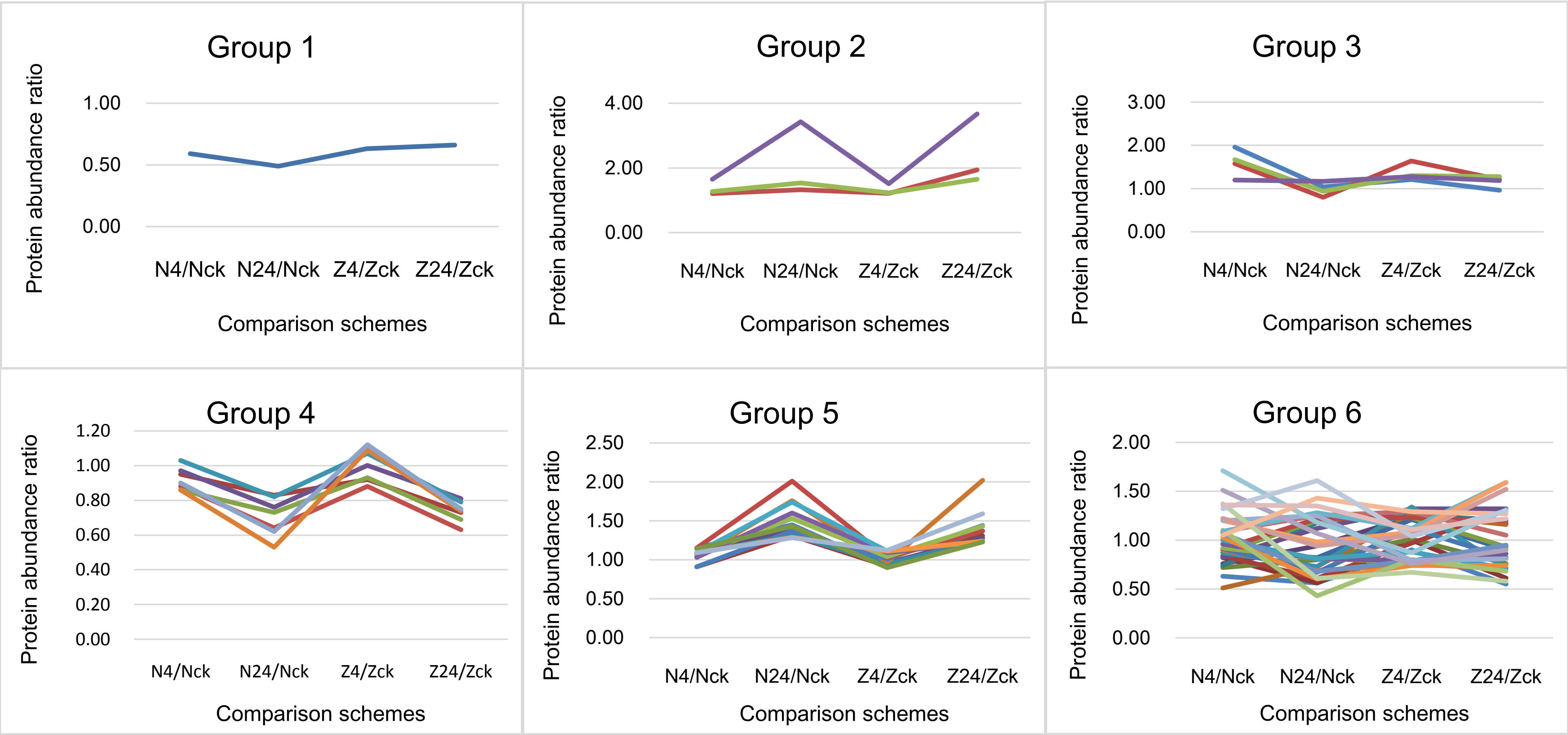

Supplementary Figure S6. Group analyses of genotype-specific proteins significantly changed in abundance under salt treatment. Six groups were generated to classify differentially abundant proteins in the 3 comparison sets Zck/Nck, Z4/N4 and Z24/N24.

Supplementary Figure S6

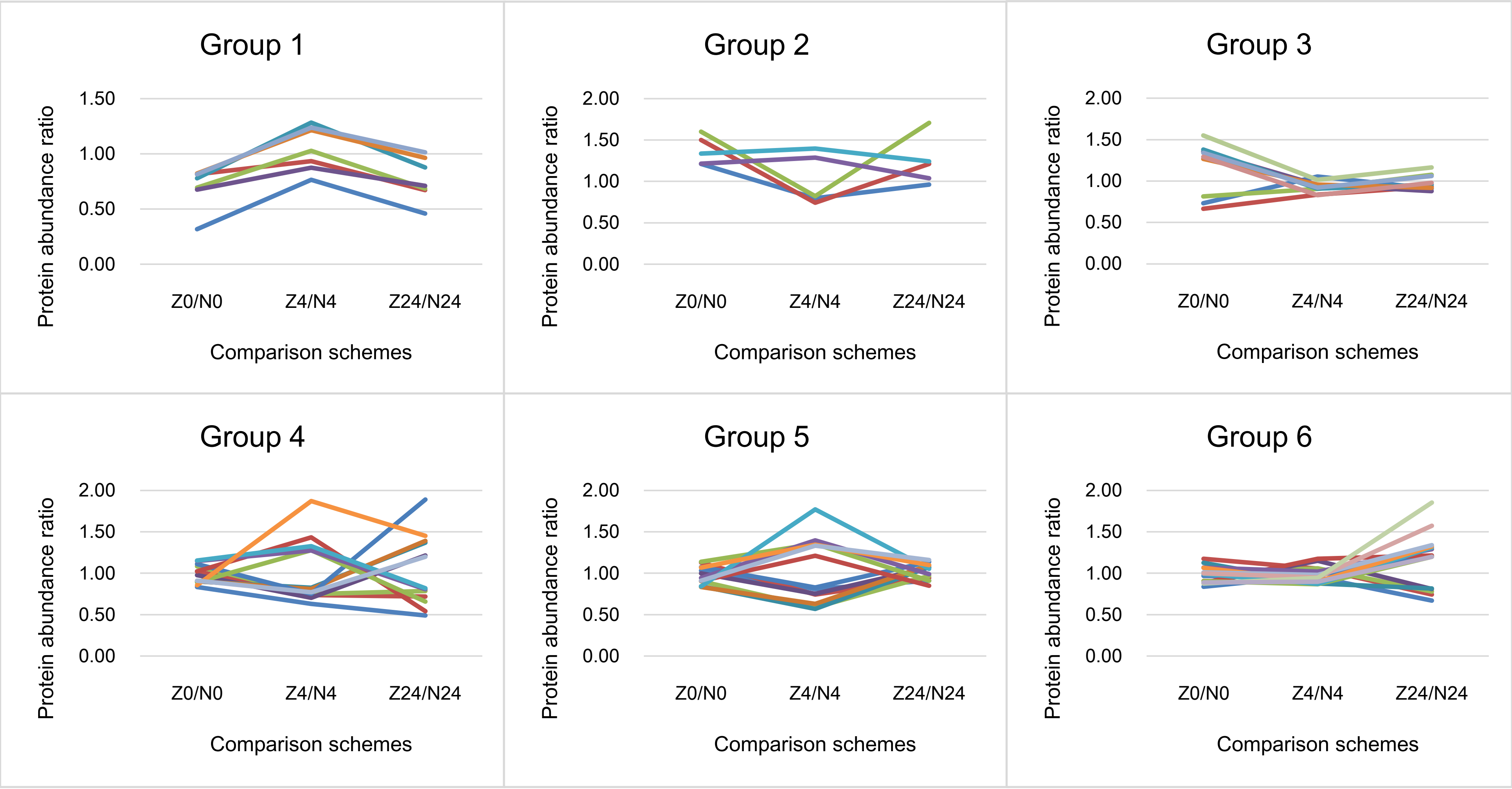

Supplementary Figure S7. The profiles of functional groups of genotype-specific DAPs. Heat map of the log 2 relative abundance of genotype-specific DAPs was created using Genesis v1.7.7 with the iTRAQ data. For each protein, the sequence description assigned with Blast2GO was provided. Proteins were grouped according to their function.The protein abundance values were scaled to range from +1 (red) to −1 (green) in the 3 comparson sets Zck/Nck, Z4/N4 and Z24/N24. Red indicated high abundant proteins and green indicated low abundant proteins compared to the control, which was set to zero (black). The details were provided in Supplementary table S5.

Supplementary Figure S7

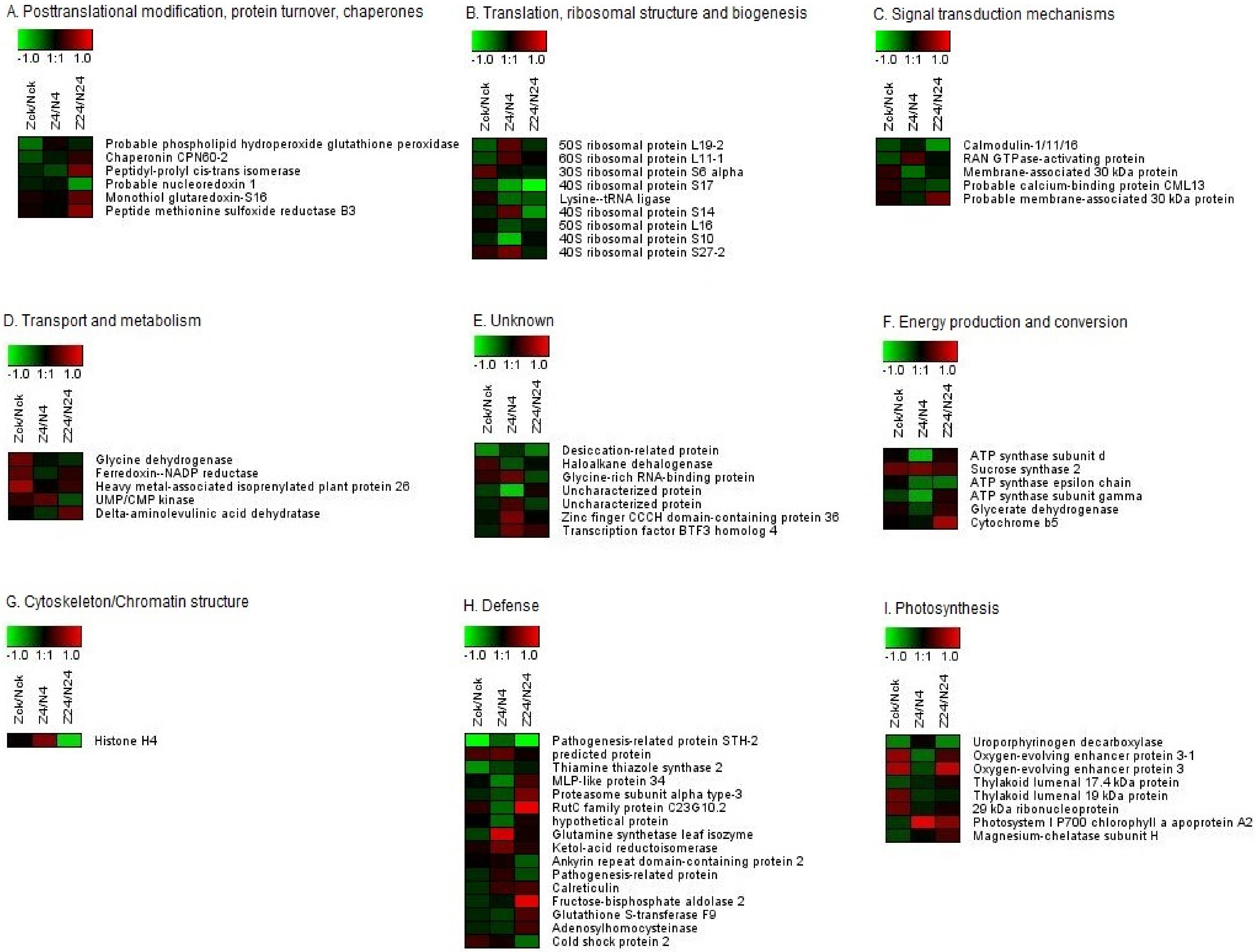

Supplementary Figure S8. The correlation of the qRT-PCR results with the abundance of 16 matched proteins. Correlation between the qRT-PCR results and protein abundance in the N4/Nck(A), Z4/Zck(B), N24/Nck(C), and Z24/ Zck (D) comparisons. The genotypes represented fitted straight trend genotypes from the data points. “r” represented the Pearson correlation coefficient.

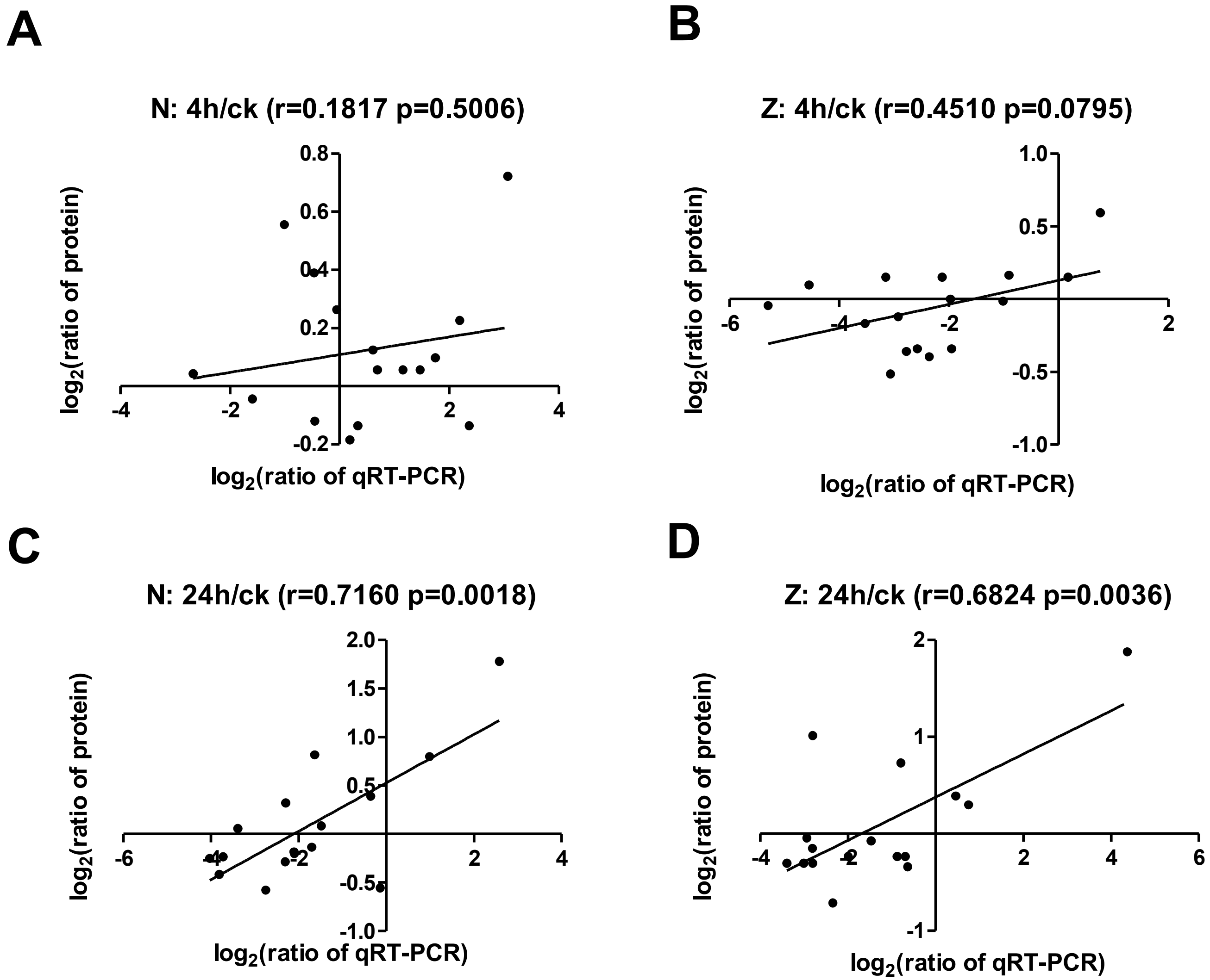
A
N: 4h/ck (r=0.1817 p=0.5006)
log2(ratio of protein)
log2(ratio of qRT-PCR)
B
Z: 4h/ck (r=0.4510 p=0.0795)
log2(ratio of protein)
log2(ratio of qRT-PCR)
C
N: 24h/ck (r=0.7160 p=0.0018)
log2(ratio of protein)
log2(ratio of qRT-PCR)
D
Z: 24h/ck (r=0.6824 p=0.0036)
log2(ratio of protein)
log2(ratio of qRT-PCR)
